# The changing etiology of liver cancer: A comparative study of global and Chinese burden trends from hepatitis dominance to metabolic risks (1990–2021) and forecasts to 2035

**DOI:** 10.1016/j.livres.2026.07.001

**Published:** 2026-07-29

**Authors:** Xue Zhou, Mingle Huang, Xiaofen Li, Minjie Zhao, Moushen Tang

**Affiliations:** a Department of Ultrasonic Medicine, Liuzhou Hospital of Guangzhou Women and Children’s Medical Center, Liuzhou, Guangxi, China; b Department of Ultrasonic Medicine, Guangzhou Medical University Affiliated Women and Children’s Medical Center, Guangzhou, China; c Department of Hepatobiliary Surgery, The Second Affiliated Hospital of Chongqing Medical University, Chongqing, China; d Centre for Lipid Research & Key Laboratory of Molecular Biology for Infectious Diseases (Ministry of Education), Institute for Viral Hepatitis, Department of Infectious Diseases, The Second Affiliated Hospital, Chongqing Medical University, Chongqing, China; e University of Pittsburgh Medical Center-Mediterranean Institute for Transplantation and Highly Specialized Therapies (UPMC-ISMETT), Palermo, Italy; f School of Life Science and Technology, ShanghaiTech University, Shanghai, China

**Keywords:** Etiology, Liver cancer, Global burden trends, Disability-adjusted life years (DALYs), Risk factor

## Abstract

**Background and aims:**

Liver cancer remains a major global health burden with a complex and shifting epidemiology. While viral hepatitis continues to drive significant morbidity, metabolic risk factors are increasingly contributing to the incidence of liver cancer. Utilizing data from the Global Burden of Disease (GBD) Study 2021, this analysis examines China’s incidence, prevalence, and age-standardized disability-adjusted life year (DALY) rates for liver cancer from 1990 to 2021, comparing these trajectories against global and regional patterns. This study further analyzed the correlations between liver cancer burden and socioeconomic development, aiming to provide evidence for targeted intervention strategies and health policy formulation.

**Methods:**

This study utilized data from the Global Burden of Disease Study 2021 to analyze the burden of liver cancer from 1990 to 2021 across 204 countries and territories. Standardized methodologies were used to estimate prevalence, incidence and DALYs. The Socio-demographic index (SDI) was applied to evaluate Socioeconomic disparities. Joinpoint regression was employed to analyze temporal trends, and age-period-cohort modeling was applied to estimate drivers of disease burden. Age-standardized rates were projected to 2035 using statistical modeling techniques.

**Results:**

Globally, the absolute number of liver cancer cases increased continuously, whereas age-standardized rates declined from 1990 to 2021. Prevalent cases increased by 113.7% (from 345,912.7 to 739,299.5), incident cases increased by 116.2% (from 244,689.4 to 529,202.5), and total DALYs grew by 70.6% (from 7,553,666.8 to 12,887,652.4); the age-standardized DALY rate (ASDR) declined with an estimated annual percentage change (EAPC) of −0.6 (95% CI –0.72 to −0.48). Hepatitis B-related DALYs decreased significantly, while non-alcoholic steatohepatitis (NASH) and alcohol-related cases increased. China achieved marked reductions in age-standardized rates: the age-standardized incidence rate (ASIR) decreased from 10.6 to 9.5 per 100,000 (EAPC –0.28, 95% CI –0.42 to −0.13), and the ASDR fell from 334.5 to 239.9 per 100,000 (EAPC –1.16, 95% CI –1.34 to −0.98), especially in hepatitis B, though metabolic-related burdens grew. Prevalent cases in China doubled (from 132,779.2 to 265,539.4) and incident cases rose by 104.0% (from 96,434.3 to 196,636.6). Gender and age disparities were evident, with males and older adults bearing higher burdens. High-income regions saw rising metabolic-related rates, whereas low- and middle-income areas faced persistent viral hepatitis burdens. Projections suggest a continued rise in metabolic-related liver cancer globally, with NASH-related rates accelerating fastest in China, underscoring the need for dual preventive strategies targeting both infection and lifestyle factors.

**Conclusions:**

The global burden of liver cancer is undergoing an epidemiological transition, marked by declining viral hepatitis-related rates but rising metabolic- and alcohol-related cases. Significant reductions in age-standardized rates were observed in China, though absolute case number increased. Socioeconomic and geographic disparities persist, with high-income regions facing growing metabolic burdens. Future trends emphasize the need for integrated strategies addressing both infectious and lifestyle-related risk factors.

## Highlights

• **Global Trends:** While absolute liver cancer counts are increasing globally, age-standardized incidence and DALY rates are declining.
• **Etiological Shift:** Liver cancer is shifting from viral hepatitis toward MASLD/NASH and alcohol-related causes.
• **China’s Paradox:** China faces a dual burden as HBV declines while metabolic risk factors increasingly drive liver cancer.
• **Future** **Outlook:** Projections through 2035 highlight the need for integrated strategies targeting infectious and metabolic liver disease.

## 1 Introduction

Liver cancer constitutes a significant global public health burden, being the sixth most commonly diagnosed cancer and the third leading cause of cancer-related mortality worldwide.^1^ Based on the Global Burden of Disease (GBD) 2021 results, the primary etiological factors for liver cancer include hepatitis B virus (HBV) infection, hepatitis C virus (HCV) infection, alcohol consumption, non-alcoholic steatohepatitis (NASH), and other causes.^2^^,^^3^ The prevalence and incidence of liver cancer are markedly elevated in developing regions of Asia and Africa. Eastern Asia exhibited the highest ASIR, followed by Northern Africa and South-Eastern Asia. Countries such as Mongolia, Egypt, and Laos faced the most severe burdens.^3^^,^^4^ China alone accounted for nearly 45% of global liver cancer cases and deaths.^5^

The GBD 2021 data reveal that the etiological landscape of liver cancer is undergoing a significant transition, especially in middle-income nations such as China.^6^ While HBV remains the dominant global driver of liver cancer disability-adjusted life years (DALYs), its age-standardized rates (ASRs) are declining globally.^7^^,^^8^ Conversely, liver cancer attributable to alcohol use and NASH shows alarming increases worldwide.^9^^,^^10^ China exemplifies this complex shift, where historically high HBV prevalence coexists with rapidly rising metabolic- and alcohol-related liver cancer risks.^7^^,^^11^ Socio-demographic index (SDI) stratification highlights this transition: Middle SDI regions, including China, exhibit declining HBV-related ASRs of liver cancer DALYs, alongside sharp rises in alcohol-related disease and NASH. High-middle SDI regions similarly show substantial NASH-related ASRs of liver cancer DALYs.^12^^,^^13^ This underscores an emerging “dual burden”, where persistent infectious causes intersect with escalating non-communicable drivers.

To address this evolving challenge, robust epidemiological intelligence is essential. The GBD study provides the most comprehensive framework for quantifying disease burden, employing DisMod-MR 2.1, a Bayesian meta-regression tool, within a systematic cascade approach.^14^ This methodology models incidence, prevalence, remission, and mortality with internal consistency, utilizing Meta-Regression-Bayesian, Regularized, Trimmed (MR-BRT) techniques to ensure cross-study comparability.^15^ Crucially, it explicitly contrasts with China’s epidemiological transition against global patterns, providing evidence critical for developing targeted national strategies for liver cancer prevention, screening, and treatment in an era of shifting etiological dominance.

## 2 Methods

### 2.1 Ethical approval

The GBD dataset is publicly available and fully anonymized. This study conducted a secondary analysis of the global burden of diseases, injuries, and risk factors based on GBD data. This study was reviewed by the Research Ethics Committee of the Women and Children’s Medical Center of Guangzhou Medical University (Approval No. 2026336A01, approval date: June 9, 2026), which determined that ethical approval and informed consent were not required.

### 2.2 Data sources and definitions

The GBD 2021 dataset provides estimates of incidence, prevalence, mortality, years of life lost (YLLs), years lived with disability (YLDs), and DALYs for 371 diseases and injuries in 204 countries and territories from 1990 to 2021.^15^^,^^16^ Data on liver cancer burden in China and globally from 1990 to 2021 were extracted via the Global Health Data Exchange (GHDx). Detailed methodological descriptions are provided in the GBD 2021 capstone publications.^14^, ^15^, ^16^

### 2.3 The socio-demographic index (SDI)

The SDI, developed within the Global Burden of Disease framework, is a composite indicator of population development status that is associated with health outcomes. It is calculated as the geometric mean of three normalized components (each scaled 0–1): the total fertility rate among women under 25 (TFU25), average years of education among individuals aged 15 and older (EDU15+), and lag-distributed income (LDI) per capita.^14^^,^^17^ Theoretically, an SDI value of 0 indicates the lowest level of development relevant to health, whereas a value of 1 reflects the highest. In GBD 2021, each of the 204 countries and territories was categorized based on its respective SDI value.^15^

### 2.4 Statistical analysis

In this study, the Estimated Annual Percentage Change (EAPC) was employed to quantify temporal trends in ASRs of liver cancer prevalence, incidence, and DALYs.^18^ The 95% uncertainty intervals (UIs) were demonstrated for each estimated quantity. Segmented regression analysis was performed using Joinpoint software (version 5.1.0.0) to quantify temporal trends in incidence, prevalence, and DALY rates for liver cancer in China and globally between 1990 and 2021.^19^ This method employs Lerman’s grid search algorithm to detect significant joinpoints where trend slopes change significantly. Statistical significance was evaluated using permutation tests with Monte Carlo simulation and Bonferroni correction.^20^ The annual percent change (APC) for each segment was calculated as the geometric mean.^21^ Both indices were calculated using the World Health Organization’s Health Equity Assessment Toolkit (HEAT), implemented in R software (version 4.5.1).

Projections of liver cancer prevalence through 2035 were generated using a multilevel logit-transformed model based on GBD 2021 data:

$$E\left[\text{logit}\left(Y_{l,a,s,y}\right)\right]=\beta_{1}SDI_{l,y}+\alpha_{l,a,s}$$

In the model, Y denotes the location-(l), age-(a), and sex-(s) specific prevalence in year (y), *β*_*1*_ represents the fixed coefficient for the SDI trajectory, and *α*_*l,a,s*_ constitutes the random intercepts. Projections of SDI incorporated recency-weighted historical trends.^14^ The model was calibrated by benchmarking 2021 forecasts against empirical GBD estimates, followed by extrapolation to 2035.

## 3 Results

### 3.1 Prevalence, incidence and DALYs of the burden of liver cancer in China and worldwide

Between 1990 and 2021, the global absolute burden of liver cancer demonstrated a substantial increase. Prevalent cases rose by 113.7%, from 345,912.7 (95% UI 299,826.7–376,632.3) to 739,299.5 (673,114.2–821,948.2), while incident cases increased by 116.2%, from 244,689.4 (224,795.5–268,549.0) to 529,202.5 (480,339.5–593,849.1). Total DALYs grew by 70.6%, reaching 12,887,652.4 (11,673,532.6–14,472,228.0) in 2021 compared to 7,553,666.8 (6,897,511.3–8,296,181.5) in 1990. Despite these absolute surges, global age-standardized rates remained relatively stable: the age-standardized prevalence rate (ASPR) moved from 7.8 (6.9–8.4) to 8.7 (7.9–9.7) per 100,000 (EAPC 0.30 [95% CI 0.15 to 0.44]), the age-standardized incidence rate (ASIR) remained largely unchanged (EAPC 0.03 [–0.08 to 0.15]), and the age-standardized DALY rate (ASDR) exhibited a modest decline (EAPC –0.60 [–0.72 to −0.48]) (Table 1, Fig. 1). China exhibited a distinct epidemiological divergence. Although absolute burden increased—with prevalent cases rising by 100.0% from 132,779.2 (108,924.1–155,563.9) to 265,539.4 (212,435.0–331,148.9) and incident cases rising by 104.0%–196,636.6 (158,273.1–243,557.7)—significant declines or stability were observed in age-standardized metrics. China’s ASIR declined from 10.6 (8.9–12.4) to 9.5 (7.7–11.8) per 100,000 (EAPC –0.28 [–0.42 to −0.13]), while the ASDR fell significantly from 334.5 (281.1–393.1) to 239.9 (192.0–299.4) per 100,000 (EAPC –1.16 [–1.34 to −0.98]) (Table 1, Fig. 1).

**Table 1:** Global and regional trends in liver cancer burden: prevalence, incidence, and disability-adjusted life years (1990–2021).

Location	1990 (95% UI)		2021 (95% UI)		EAPC (95% CI)
	Number	ASR	Number	ASR	
**Prevalence**					
Global	345,912.7 (299,826.7–376,632.3)	7.8 (6.9–8.4)	739,299.5 (673,114.2–821,948.2)	8.7 (7.9–9.7)	0.30 (0.15–0.44)
China	132,779.2 (108,924.1–155,563.9)	13.5 (11.2–15.8)	265,539.4 (212,435–331,148.9)	13.3 (10.7–16.4)	−0.10 (−0.28 to 0.09)
Low SDI	27,803.6.0 (17,093.4–34,907.0)	7.8 (5.7–10.0)	42,599.3 (31,334.5–55,640.2)	6.0 (4.9–7.7)	−1.01 (−1.09 to −0.93)
Low-middle SDI	39,208.9 (27,884.9–45,654.9)	4.6 (3.8–5.5)	74,893.9 (66,170.8–84,278.5)	4.7 (4.2–5.3)	0.10 (0.08–0.12)
Middle SDI	113,375.1 (96,515.8–128,049.3)	8.7 (7.6–9.8)	231,125.3 (198,437.2–276,537.5)	8.4 (7.3–10.0)	−0.04 (−0.21 to 0.12)
High-middle SDI	78,560.1 (68,889–88,621.5)	7.7 (6.7–8.6)	155,296.4 (132,500.8–184,366.7)	8.4 (7.2–10.0)	0.16 (−0.01 to 0.33)
High SDI	86,780.2 (82,354.3–90,714.5)	8.3 (7.9–8.7)	235,057.5 (213,883.8–249,189.8)	12.2 (11.3–12.9)	1.02 (0.63–1.42)
Andean Latin America	888.1 (643.8–1082.9)	3.2 (2.6–3.7)	1688.1 (1309.6–2130.8)	2.8 (2.2–3.5)	−0.52 (−0.78 to −0.26)
Australasia	596.3 (552.2–643.3)	2.7 (2.5–2.9)	4497.3 (4028.3–5013.4)	9.7 (8.7–10.8)	4.52 (4.22–4.82)
Caribbean	632.1 (546.2–715.2)	2.2 (2.0–2.5)	1095.1 (945.6–1261.2)	2.1 (1.8–2.4)	−0.55 (−0.82 to −0.29)
Central Asia	4834 (4176.8–5468.6)	8.8 (7.9–9.9)	6483.1 (5503.5–7587.7)	7.4 (6.3–8.6)	−0.74 (−0.82 to −0.66)
Central Europe	4940.0 (4549.9–5350.0)	3.4 (3.2–3.7)	6590.4 (5916.7–7338.5)	3.2 (2.9–3.6)	−0.24 (−0.40 to −0.08)
Central Latin America	3848.2 (2922.0–4226.6)	3.4 (3.0–3.6)	7993.0 (7128.5–8905.6)	3.2 (2.9–3.6)	−0.02 (−0.26 to 0.23)
Central Sub-Saharan Africa	3031.3 (1402.8–5004.1)	8.4 (4.1–16.3)	4051.0 (1901.6–8229.6)	5.5 (2.6–11.3)	−1.67 (−1.79 to −1.55)
East Asia	136,980.9 (112,967.7–159,079.5)	13.4 (11.2–15.6)	275,114.3 (222,236.6–340,133.4)	13.3 (10.8–16.3)	−0.05 (−0.22 to 0.11)
Eastern Europe	6380.8 (6068.8–6692.9)	2.6 (2.4–2.7)	8841.8 (8195.2–9557.3)	3 (2.8–3.2)	0.45 (0.26–0.65)
Eastern Sub-Saharan Africa	9417.0 (5664.6–12,443.6)	7.2 (5.6–9.3)	14,459.4 (9980–20,950.1)	6 (4.5–7.9)	−0.87 (−1.01 to −0.72)
High-income Asia Pacific	53,965.8 (49,694.2–57,925.3)	26.4 (24.3–28.3)	108,802.6 (94,715.4–119,758.5)	25.3 (22.4–27.9)	−0.65 (−1.11 to −0.18)
High-income North America	11,004.3 (10,614.7–11,253.9)	3.4 (3.3–3.5)	53,050.3 (49,942.5–55,144.4)	9.2 (8.7–9.5)	3.34 (3.01–3.67)
North Africa and Middle East	12,678.4 (9108.8–16,035.8)	5.6 (4.3–7.5)	28,482.4 (24,243.8–32,966.3)	5.7 (4.9–6.6)	0.23 (0.15–0.32)
Oceania	184.1 (113.9–352.9)	4.8 (3.1–9.4)	348.6 (228.5–632.0)	3.7 (2.5–6.7)	−0.99 (−1.11 to −0.86)
South Asia	22,968.6 (15,572.8–28,235.5)	2.7 (2.2–3.1)	52,275.8 (45,780.1–58,685.8)	3.3 (2.9–3.7)	0.70 (0.66–0.74)
Southeast Asia	26,447.1 (22,411.3–30,316.8)	8.3 (7.2–9.4)	53,637.8 (42,303.0–69,356.3)	7.7 (6.0–9.8)	−0.35 (−0.42 to −0.28)
Southern Latin America	414.8 (367.7–471.3)	0.9 (0.8–1.0)	1682.0 (1514.9–1852.7)	2.0 (1.8–2.2)	3.25 (3.06–3.44)
Southern Sub-Saharan Africa	2093.6 (1304.3–3180.8)	6.2 (3.9–9.6)	5374.7 (4514.7–6299.3)	8.2 (7.0–9.6)	0.30 (−0.29 to 0.89)
Tropical Latin America	2261.9 (1838.5–2489.2)	2.0 (1.8–2.2)	5227.1 (4917.6–5511.4)	2.1 (1.9–2.2)	0.59 (0.36–0.81)
Western Europe	24,257.7 (23,274.5–25,211.1)	4.6 (4.4–4.8)	71,755.0 (66,420.6–76,176.1)	8.9 (8.3–9.4)	2.33 (2.07–2.60)
Western Sub-Saharan Africa	18,087.8 (10,802.3–24631.6)	14.0 (9.3–19.6)	27,849.9 (21,406.4–34,130.7)	9.9 (8.1–11.7)	−1.35 (−1.45 to −1.25)
**Incidence**					
Global	244,689.4 (224,795.5–268,549.0)	5.9 (5.4–6.5)	529,202.5 (480,339.5–593,849.1)	6.2 (5.6–6.9)	0.03 (−0.08 to 0.15)
China	96,434.3 (80,970.6–113,768.7)	10.6 (8.9–12.4)	196,636.6 (158,273.1–243,557.7)	9.5 (7.7–11.8)	−0.28 (−0.42 to −0.13)
Low SDI	16,451.4 (12,329.6–21,500.6)	6.6 (4.8–8.8)	28,787.1 (23,443.5–36,441.3)	5.2 (4.3–6.6)	−0.96 (−1.05 to −0.87)
Low-middle SDI	25,180.6 (21,808.7–30,490.3)	3.8 (3.2–4.7)	60,181.4 (54,564.7–66,690.6)	4.0 (3.7–4.5)	0.21 (0.19–0.22)
Middle SDI	80,013.9 (70,274.2–90,052)	7.0 (6.2–7.9)	179,743.3 (155,621.4–213,998.0)	6.6 (5.7–7.8)	−0.09 (−0.22 to 0.03)
High-middle SDI	60,675.7 (53,597.6–68,149.7)	6 (5.3–6.7)	117,674.1 (101,084.2–138,477.3)	6.1 (5.2–7.2)	−0.05 (−0.20 to 0.11)
High SDI	62,216.8 (58,839.7–65,523.1)	5.8 (5.5–6.1)	142,545.8 (129,643.9–151,308.8)	6.9 (6.4–7.3)	0.24 (−0.04 to 0.52)
Andean Latin America	560.5 (495.3–651.3)	2.6 (2.3–3.0)	1483.3 (1146.9–1863.5)	2.5 (1.9–3.2)	−0.19 (−0.44 to 0.06)
Australasia	452.7 (417.5–488.9)	2.0 (1.8–2.1)	2946.9 (2626.8–3286.4)	5.8 (5.2–6.4)	3.61 (3.44–3.78)
Caribbean	521 (475.3–582.6)	2.0 (1.8–2.2)	966.9 (841.7–1105.3)	1.8 (1.6–2.1)	−0.66 (−0.93 to −0.39)
Central Asia	3656.3 (3277.1–4034.8)	7.5 (6.7–8.3)	5676.5 (4818.6–6630.6)	6.8 (5.8–7.9)	−0.47 (−0.56 to −0.37)
Central Europe	4442.0 (4098.4–4816.3)	3.0 (2.7–3.2)	6276.6 (5657.8–6980.1)	2.9 (2.6–3.2)	−0.18 (−0.32 to −0.04)
Central Latin America	2490.8 (2407.6–2567.9)	2.9 (2.8–3)	7066.3 (6407.2–7793.4)	2.9 (2.6–3.2)	0.10 (−0.16 to 0.37)
Central Sub-Saharan Africa	1843.2 (908.4–3593.1)	7.4 (3.5–15.4)	2971.5 (1443.4–6042.6)	5.1 (2.4–10.6)	−1.52 (−1.65 to −1.40)
East Asia	99,568.9 (84,081.3–117,027.0)	10.5 (8.9–12.3)	203,597 (165,109.9–250,430.7)	9.5 (7.8–11.7)	−0.24 (−0.37 to −0.11)
Eastern Europe	4866.7 (4643.4–5093.6)	1.8 (1.7–1.8)	7936.1 (7340.7–8551.9)	2.3 (2.2–2.5)	0.94 (0.71–1.17)
Eastern Sub-Saharan Africa	5121.8 (4094.3–6583.8)	6.1 (4.9–7.9)	9727.1 (7371.9–12,831.3)	5.4 (4.1–7.0)	−0.77 (−0.95 to −0.59)
High-income Asia Pacific	36,301.7 (32,997.0–39,318.3)	17.6 (16.1–19.1)	58,469 (50,924.3–64,562.0)	12.6 (11.3–14.0)	−1.75 (−2.12 to −1.37)
High-income North America	8002.0 (7610.1–8219.7)	2.3 (2.2–2.4)	34,973.4 (32,429.7–36,413.9)	5.5 (5.2–5.7)	2.80 (2.60–3.00)
North Africa and Middle East	8477.3 (6754.2–11,645.9)	4.8 (3.7–6.7)	23,221.1 (19,722.4–27,037.4)	5 (4.3–5.8)	0.28 (0.20–0.36)
Oceania	140.8 (88.4–277.9)	4.4 (2.8–8.5)	271.0 (179.3–489.0)	3.3 (2.2–5.9)	−1.06 (−1.17 to −0.94)
South Asia	13,538.3 (12,285.6–14,938.3)	2.1 (1.9–2.3)	42,094.4 (37,827.3–46,445.1)	2.8 (2.5–3.1)	0.94 (0.90–0.99)
Southeast Asia	19,102.9 (16,751.7–21,836.9)	6.9 (6.1–7.9)	44,024.9 (34,718.7–56,944.1)	6.5 (5.1–8.4)	−0.33 (−0.42 to −0.24)
Southern Latin America	372.1 (332.8–420.7)	0.8 (0.7–0.9)	1537.0 (1382.5–1689.7)	1.8 (1.6–1.9)	3.16 (2.97–3.35)
Southern Sub-Saharan Africa	1582.6 (997.9–2448.1)	5.4 (3.4–8.4)	4514.6 (3849.4–5310.5)	7.5 (6.4–8.7)	0.40 (−0.21 to 1.02)
Tropical Latin America	1564.3 (1496.0–1620.9)	1.7 (1.6–1.7)	4622.4 (4337.7–4855.2)	1.8 (1.7–1.9)	0.81 (0.59–1.04)
Western Europe	20,393.7 (19,350.2–21,265.0)	3.6 (3.4–3.7)	47,853.0 (43,676.9–50,700.1)	5.3 (4.9–5.6)	1.33 (1.18–1.47)
Western Sub-Saharan Africa	11,689.7 (7830.3–16,709.9)	12.0 (8.0–17.4)	18,973.3 (15,420.6–22,514.3)	8.7 (7.3–10.2)	−1.28 (−1.39 to −1.17)
**Disability-adjusted life years (DALYs)**					
Global	7,553,666.8 (6,897,511.3–8,296,181.5)	172.9 (157.8–190.2)	12,887,652.4 (11,673,532.6–14,472,228.0)	149.3 (135.2–167.5)	−0.60 (–0.72 to −0.48)
China	3,294,864.3 (2,763,029.0–3,879,589.0)	334.5 (281.1–393.1)	4,890,023.0 (3,905,088.6–6,124,599.2)	239.9 (192.0–299.4)	−1.16 (−1.34 to −0.98)
Low SDI	569,334.3 (430,546.2–728,122.7)	193.6 (142.8–255.8)	964,541.7 (772,939.2–1,243,934.3)	151.1 (122.4–192.8)	−1.01 (−1.11 to −0.91)
Low-middle SDI	848,140.2 (745,554.9–1,001,883.5)	112.7 (97.6–137.5)	1,839,381.9 (1,651,208.7–2,045,499.9)	116.4 (104.9–129.0)	0.06 (0.04–0.09)
Middle SDI	2,719,978.9 (2,384,909.3–3,065,583.6)	216.8 (190.1–243.7)	4,746,027.9 (4,107,709.7–5,643,717.9)	169.7 (147.4–200.9)	−0.75 (−0.87 to −0.63)
High-middle SDI	1,910,910.7 (1,671,066.1–2,172,671.8)	184.4 (161.6–209.2)	2,877,560.1 (2,446,560.3–3,427,883.8)	152.6 (129.5–181.8)	−0.86 (−1.04 to −0.67)
High SDI	1,500,946.1 (1,410,509.0–1,595,214.5)	143.2 (134.5–152.4)	2,453,150.1 (2,289,462.8–2,587,416.4)	127.9 (120.6–134.4)	−0.64 (−0.87 to −0.41)
Andean Latin America	18,419.5 (16,211.1–21,143.2)	73.8 (65.3–85.8)	39,834.4 (30,595.9–50,171.6)	65.8 (50.6–82.9)	−0.57 (−0.84 to −0.30)
Australasia	11,404.8 (10,571.8–12,308.5)	50.0 (46.4–54.0)	58,668.3 (52,503.7–65,202.8)	122.0 (109.8–135.5)	2.90 (2.80–3.00)
Caribbean	14,774.2 (13,482.0–16,466.8)	53.8 (49.0–60.3)	25,728.8 (22,106.7–29,711.3)	48.4 (41.5–55.9)	−0.66 (−0.94 to −0.38)
Central Asia	114,792.6 (103,024.4–126,751.9)	222.0 (199.4–246.2)	167,974.3 (142,229.5–197,862.3)	188.8 (160.0–221.6)	−0.79 (−0.88 to −0.71)
Central Europe	122,248.4 (112,650.1–132,286.4)	82.2 (75.7–88.8)	152,063.6 (136,011.5–169,478.7)	73.7 (66.0–82.1)	−0.40 (−0.58 to −0.22)
Central Latin America	76,751.4 (74,643.7–78,919.1)	78.5 (76.1–80.7)	185,645.8 (167,689.4–204,995.0)	73.6 (66.5–81.2)	−0.15 (−0.45 to 0.16)
Central Sub-Saharan Africa	64,169.3 (32,287.7–120,450.0)	215.6 (103.9–436.5)	99,409.4 (47,597.8–209,360.4)	143.5 (69.1–297.6)	−1.63 (−1.74 to −1.51)
East Asia	3,400,330.2 (2,860,133.3–3,991,707.5)	332.6 (280.3–389.7)	5,063,799.9 (4,074,239.5–6,285,122.1)	239.8 (193.3–296.8)	−1.13 (−1.29 to −0.96)
Eastern Europe	143,783.8 (137,825.9–150,043.0)	53.5 (51.3–55.7)	208,185.0 (192,394.7–225,655.5)	64.4 (59.7–69.7)	0.67 (0.38–0.96)
Eastern Sub-Saharan Africa	180,117.4 (145,195.9–230,083.4)	176.6 (140.9–229.1)	325,428.2 (242,047.5–438,427.3)	150.4 (114.0–198.6)	−0.84 (−1.02 to −0.65)
High–income Asia Pacific	877,092.5 (784,034.1–967,492.7)	422.3 (377.2–465.7)	839,314.4 (750,067.4–931,624.8)	205.8 (185.3–229.3)	−2.88 (−3.26 to −2.50)
High-income North America	171,185.2 (164,907.4–174,995.0)	51.9 (50.1–53.0)	646,402.5 (610,397.8–670,701.1)	106.0 (100.6–110.0)	2.43 (2.26–2.60)
North Africa and Middle East	272,364.6 (221,559.0–361,798.3)	134.8 (107.3–185.1)	673,287.3 (567,573.7–783,681.4)	134.8 (114.1–156.6)	0.09 (0.02–0.16)
Oceania	4794.2 (2982.7–9511.2)	129.2 (81.4–256.2)	8992.0 (5879.1–16,559.3)	96.4 (63.7–175.1)	−1.09 (−1.20 to −0.97)
South Asia	469,587.1 (426,811.7–516,427.1)	63.8 (57.8–70.7)	1,274,599.1 (1,146,363.8–1,411,896.8)	79.7 (71.8–88.0)	0.71 (0.67–0.75)
Southeast Asia	631,449.7 (558,091.5–719,439.4)	206.8 (182.4–236.9)	1,287,277.3 (1,007,128.9–1,686,531.1)	179.7 (141.2–234.3)	−0.58 (−0.67 to −0.49)
Southern Latin America	10,126.3 (9024.0–11,519.9)	21.5 (19.2–24.5)	37,431.2 (33,708.8–41,091.4)	44.0 (39.7–48.3)	2.94 (2.70–3.18)
Southern Sub–Saharan Africa	53,466.1 (33,947.5–82,759.3)	162.4 (102.2–251.6)	144,312.1 (121,227.1–170,750.5)	218.2 (185.2–257.2)	0.29 (−0.38 to 0.97)
Tropical Latin America	49,477.0 (47,586.5–51,453.7)	47.0 (45.1–48.8)	125,606.1 (119,005.4–131,666.1)	48.6 (46.0–51.0)	0.72 (0.49–0.96)
Western Europe	470,612.5 (451,834.6–489,447.0)	86.2 (83.1–89.4)	881,873.6 (819,766.3–929,895.9)	106.1 (99.8–111.4)	0.68 (0.56–0.79)
Western Sub–Saharan Africa	396,720.1 (268,095.8–554,986.0)	357.0 (236.1–511.7)	641,819.0 (515,895.7–773,751.2)	249.9 (204.2–298.7)	−1.38 (−1.50 to −1.27)

Abbreviations: CI, confidence interval; SDI, the Socio-demographic index; UI, uncertainty interval.

**Fig. 1:**
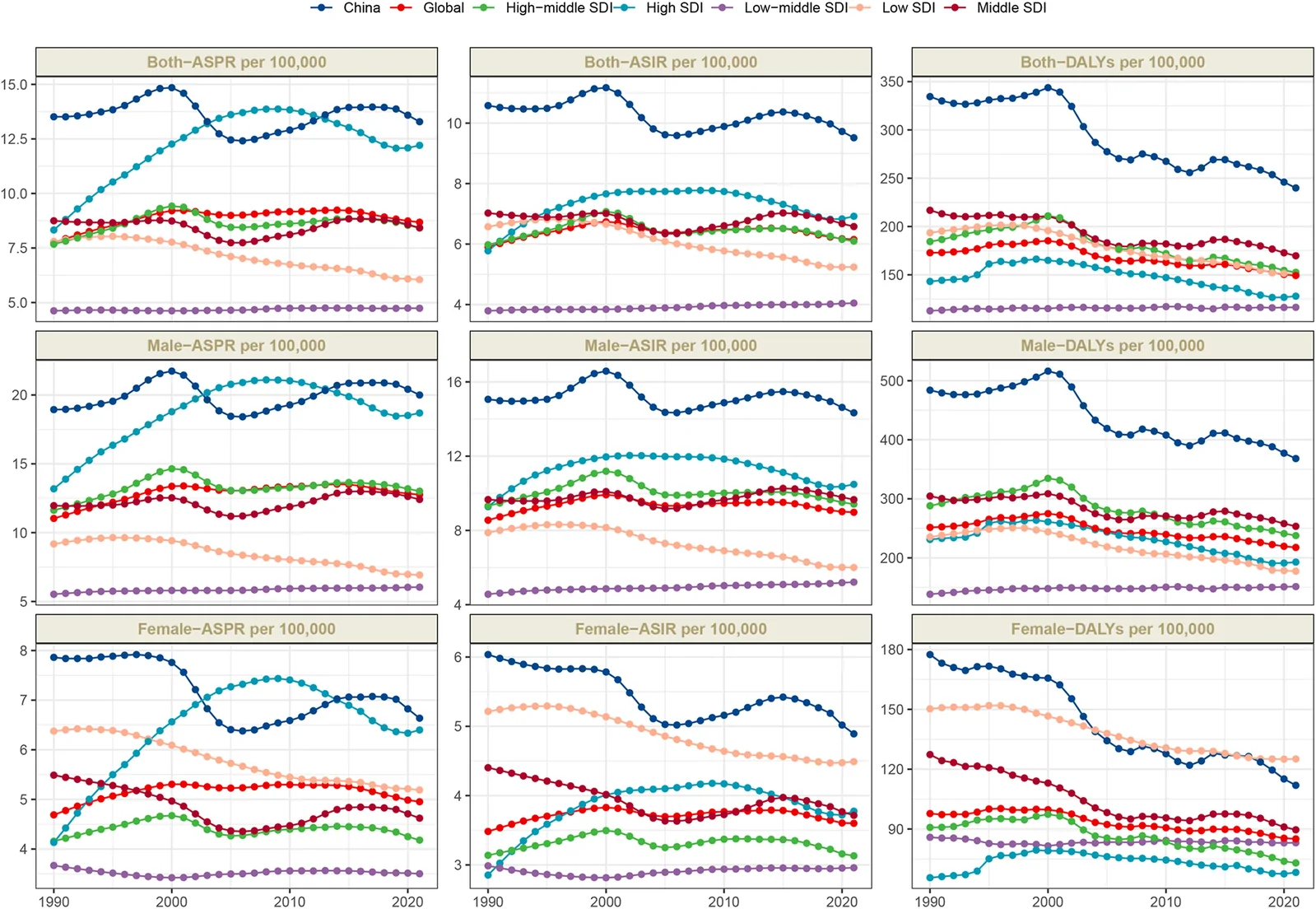
**Trends in liver cancer prevalence, incidence and disability-adjusted life-years from 1990 to 2021.** The trends are stratified by sex (both sexes, males, and females) and metrics: age-standardized prevalence rate (ASPR) per 100,000 population; age-standardized incidence rate (ASIR) per 100,000 population; and age-standardized DALY rate (ASDR) per 100,000 population. Abbreviations: DALYs, disability-adjusted life years; SDI, Socio-demographic index.

Analysis by SDI revealed that High SDI regions experienced the most rapid increase in age-standardized burden, with an ASPR EAPC of 1.02 (0.63–1.42), while Low SDI regions achieved the most significant reductions in both ASPR (EAPC –1.01 [–1.09 to −0.93]) and ASDR (EAPC –1.01 [–1.11 to −0.91]). Regionally, High-income Asia Pacific recorded a major reduction in DALY rates (ASDR EAPC –2.88 [–3.26 to −2.50]). In 2021, the highest age-standardized prevalence was observed in the High-income Asia Pacific region (ASPR 25.3 [22.4–27.9]), while the lowest was in Southern Latin America (ASPR 2.0 [1.8–2.2]) (Table 1, Fig. 1, Figs. S1 and S2).

### 3.2 Global trends of liver cancer by country

In 2021, significant geographic disparities in the burden of liver cancer were observed globally, with age-standardized rates showing marked variation across countries. The highest age-standardized rates were recorded in Mongolia, which exhibited an ASPR of 76.6 (95% UI 58.8–97.9) per 100,000 population, an ASIR of 74.6 (57.2–94.5) per 100,000, and an ASDR of 1993.2 (1531.4–2547.2) per 100,000. Conversely, the lowest burden was observed in Morocco, with an ASPR of 0.6 (0.4–0.8) per 100,000, an ASIR of 0.5 (0.4–0.6) per 100,000, and an ASDR of 14.3 (10.1–18.5) per 100,000 (Tables S1–S3).

### 3.3 Gender disparities in the burden of liver cancer in different age groups worldwide

In 2021, the global burden of liver cancer DALYs exhibited distinct patterns when stratified by etiology, sex, and age. HBV infection remained a primary driver, with absolute DALYs peaking in middle-aged adults (50–54 years). This etiology demonstrated the most significant sex disparity across all causes, with a male-to-female DALY ratio exceeding 3:1 (Fig. 2B). In contrast, the burden attributable to NASH showed a progressive, age-dependent trajectory; while absolute counts were substantial in middle age (55–69 years), the highest ASDR rates were observed in the older cohorts (75+ years) (Fig. 2E).

**Fig. 2:**
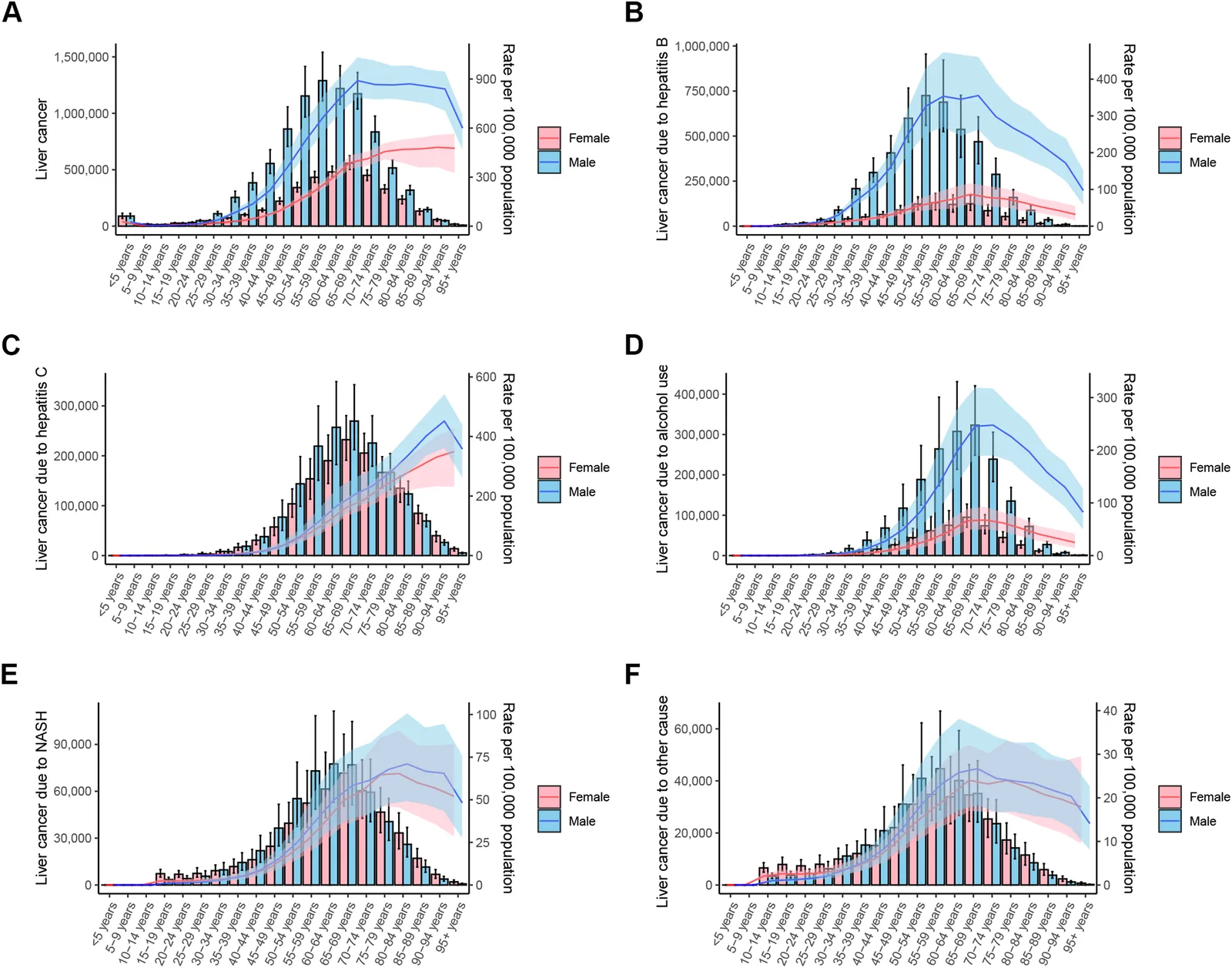
**Theglobal** a**ge- and** s**ex-**s**pecific disability-adjusted life years (DALYs) of Liver Cancer in 2021 by Etiology.** The distinct etiological categories: all-cause **(A)**, hepatitis B **(B)**, hepatitis C **(C)**, alcohol-related **(D)**, non-alcoholic steatohepatitis (NASH)-related **(E)**, and other causes **(F)**.

### 3.4 Sex, age and etiology in the global epidemiology of liver cancer in 2021

In 2021, the global epidemiology of liver cancer was characterized by a pronounced male-to-female ratio of approximately 2:1, with a peak burden concentrated in the 70–74 years age group across prevalence, incidence, and DALYs (Fig. 3). The etiological landscape transitioned significantly across the life course: hepatitis B remained a primary driver in younger cohorts (<50 years), while the proportional contribution of alcohol use and NASH increased progressively with age, dominating the disease profile in elderly populations (70+ years) (Fig. 3).

**Fig. 3:**
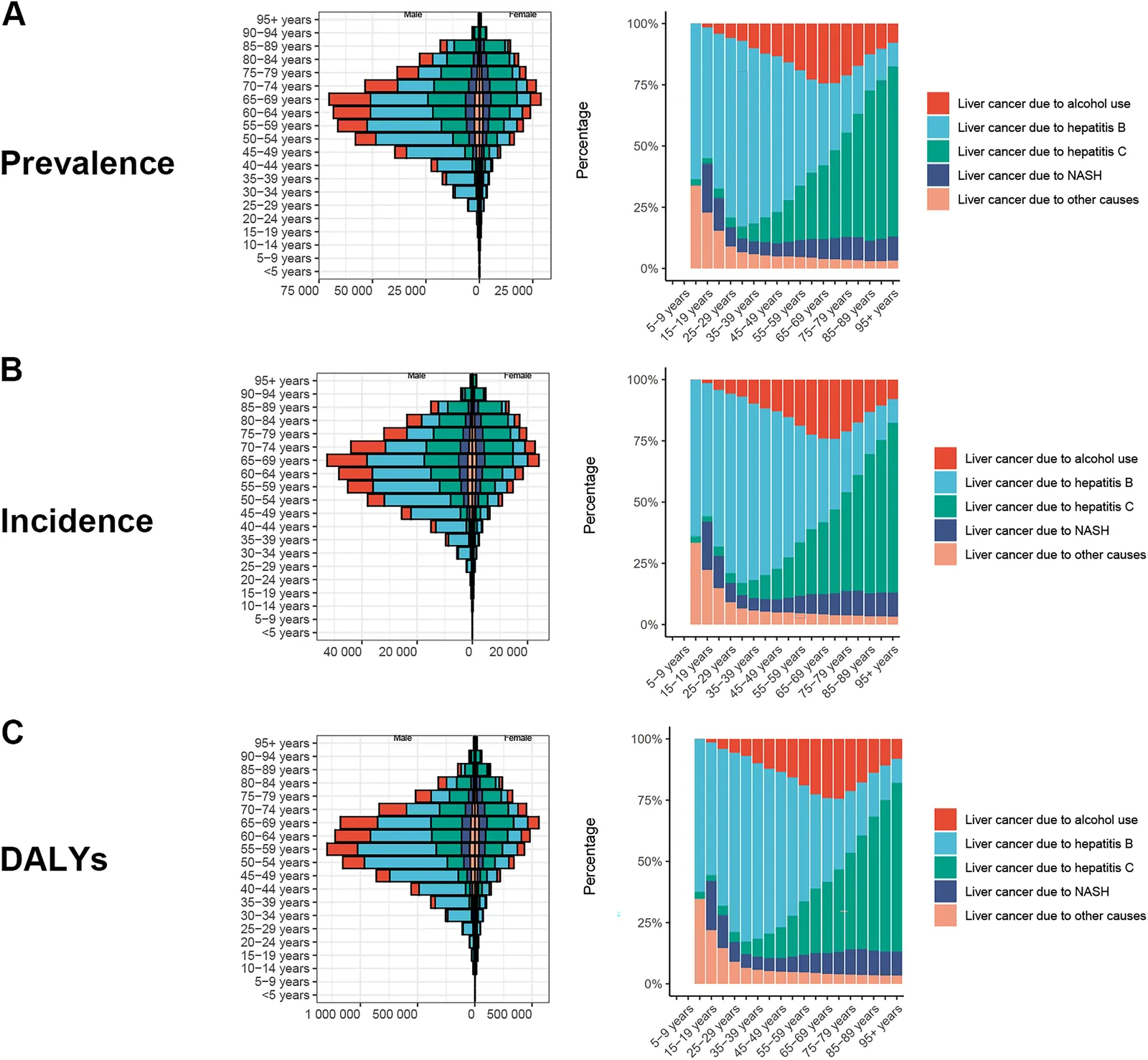
**Sex and age stratification in the global epidemiology of liver cancer etiology in 2021. (A)** Prevalence of liver cancer distribution and proportion by etiology. **(B)** Incidence of liver cancer distribution and proportion by etiology. **(C)** DALYs of liver cancer distribution and proportion by etiology. Abbreviations: DALYs, disability-adjusted life years; NASH, non-alcoholic steatohepatitis.

### 3.5 The global epidemiological patterns of HCC

The global epidemiological patterns of liver cancer in 2021 exhibit marked regional heterogeneity across prevalence, incidence, and DALYs. HBV remains the dominant driver globally, accounting for 35%–50% of cases, with the highest absolute burden concentrated in Sub-Saharan Africa and East Asia. While incidence and DALY patterns largely mirror prevalence, reflecting the persistent impact of viral hepatitis in developing regions, a distinct transition is observed in high-income regions (North America, Western Europe), where the proportional contribution of alcohol-related HCC and NASH is significantly elevated (Fig. 4.).

**Fig. 4:**
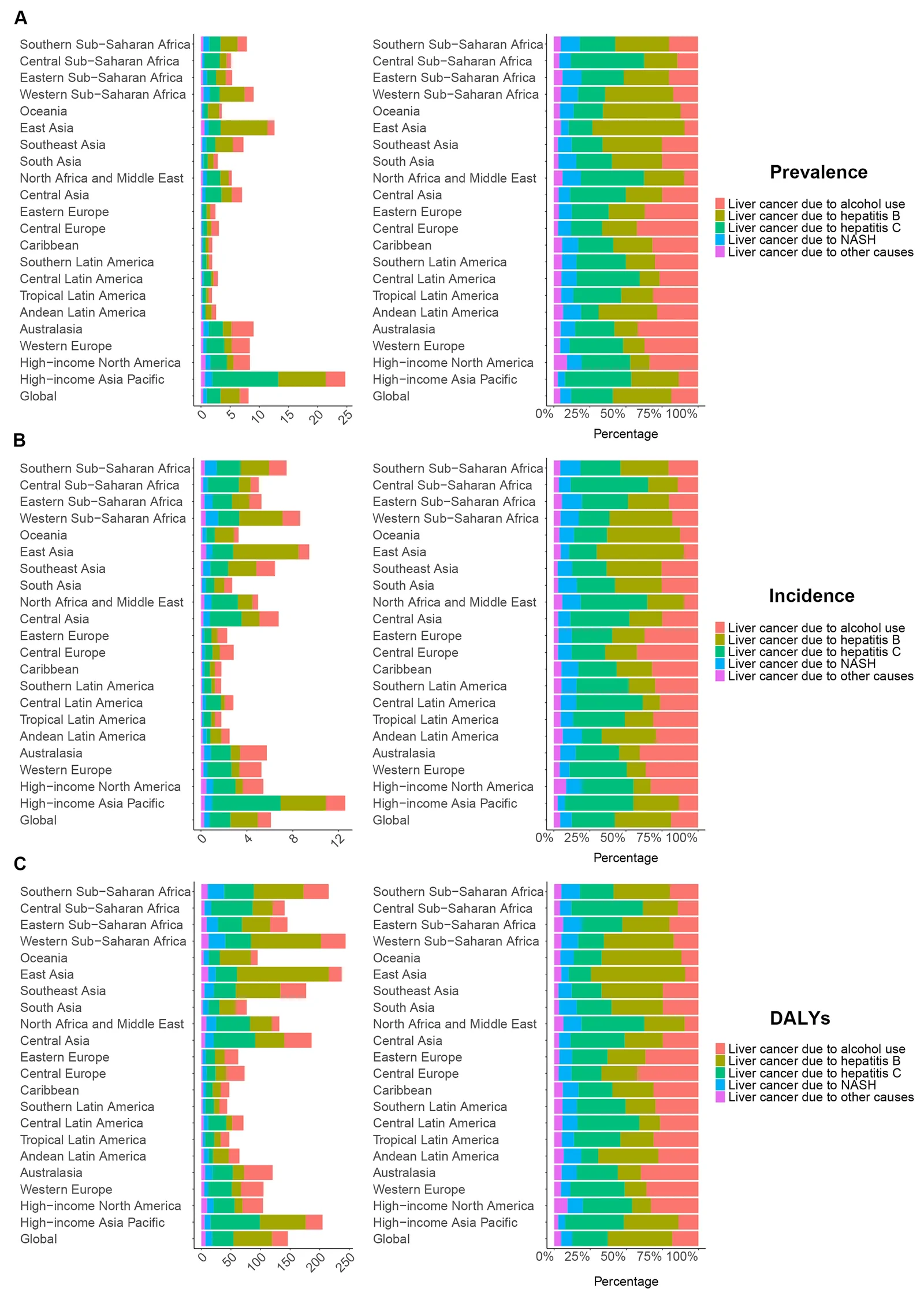
**The global epidemiological patterns of HCC.(A)** Global prevalence of liver cancer etiological distribution and proportional composition. **(B)** Incidence patterns of liver cancer etiological stratification and geographical variation. **(C)** Burden of liver cancer measured in DALYs etiological attribution and regional disparities. Abbreviations: DALYs, disability-adjusted life years; NASH, non-alcoholic steatohepatitis.

### 3.6 The global burden of liver cancer by region and etiology

The global landscape of liver cancer in 2021, as measured by prevalence, incidence, and DALY rates, reveals profound regional disparities driven by distinct etiological profiles. HBV remains the primary driver of disease burden globally, with the most severe impact concentrated in High-income Asia Pacific and East Asia, where rates consistently fall within the highest quintile across all health metrics. In contrast, HCV burden is notably elevated in Central Asia and Eastern Europe, while alcohol-related liver cancer exhibits moderate burden concentrations (0.5–0.6) primarily in Western Europe and other high-income regions (Fig. 5). Although NASH-related cases currently display a lower absolute burden (0.3–0.4) compared to viral etiologies, they show a widespread geographical distribution with disproportionately higher proportional contributions to the total disease burden in Western Europe and High-income North America (Fig. 5).

**Fig. 5:**
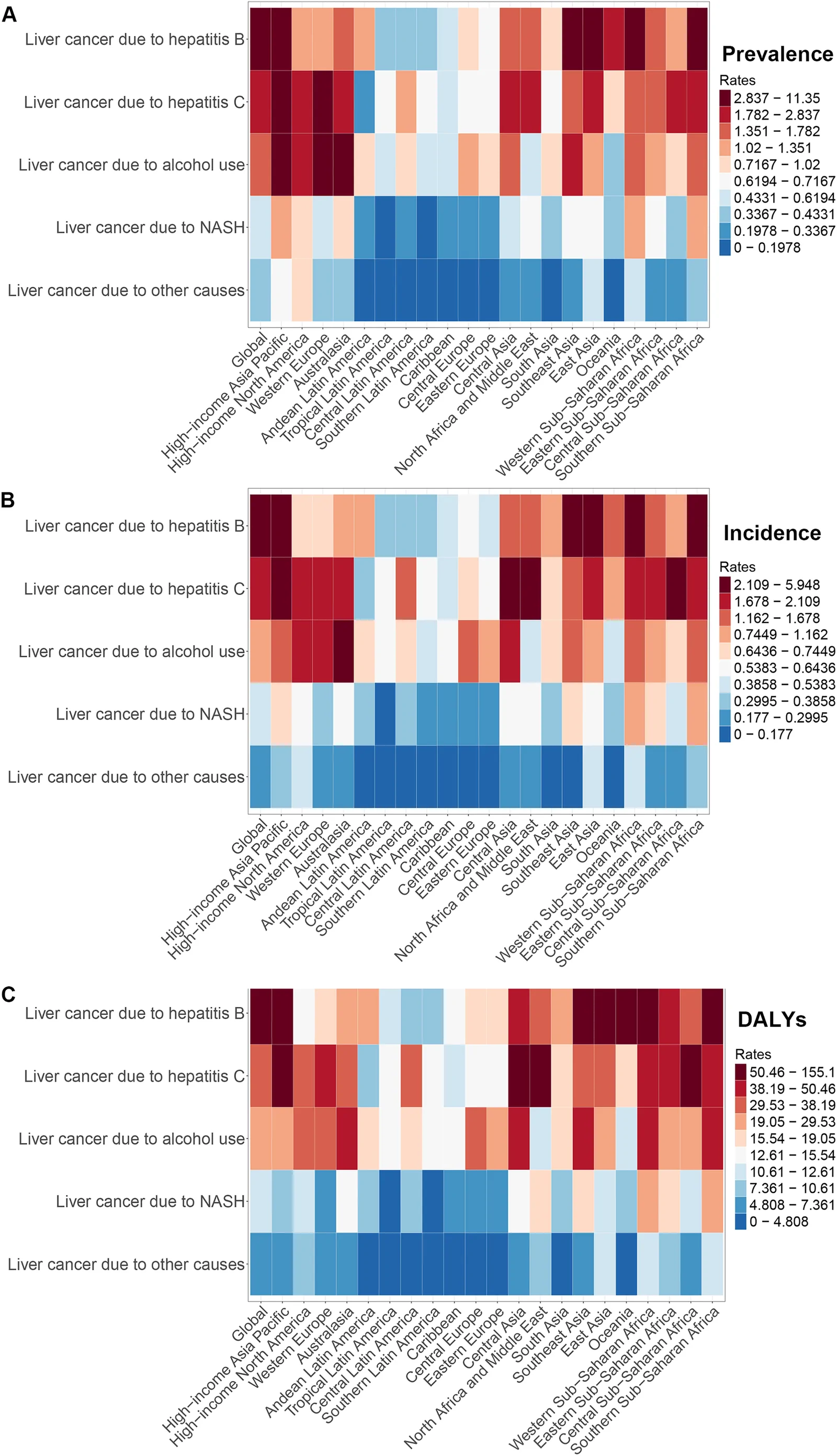
**The global burden of liver cancer by region and etiology. (A)** Global prevalence of liver cancer with etiological composition and geographical distribution. **(B)** Global incidence of liver cancer showing etiological stratification and regional variation. **(C)** Global burden of liver cancer measured in disability-adjusted life years by etiology and region. Abbreviations: DALYs, disability-adjusted life years; NASH, non-alcoholic steatohepatitis.

### 3.7 Joinpoint regression analysis of the global burden of HCC

Between 1990 and 2021, the global ASDR for liver cancer exhibited complex, nonlinear temporal patterns characterized by significant etiological divergence. While the overall ASDR for liver cancer showed a general downward trend following multiple inflection points, this decline was primarily driven by a consistent and significant reduction in Hepatitis B-associated ASDR throughout the study period (Fig. 6B). Hepatitis C-related ASDR followed a biphasic trajectory, characterized by an initial rise until the late 1990s followed by a subsequent, sustained decline (Fig. 6C). Conversely, NASH-associated ASDR demonstrated the most substantial and concerning increasing trend among all etiological classifications, reflecting the growing global impact of metabolic risk factors (Fig. 6D). Alcohol-related liver cancer mortality remained relatively stable with only minor fluctuations (Fig. 6E).

**Fig. 6:**
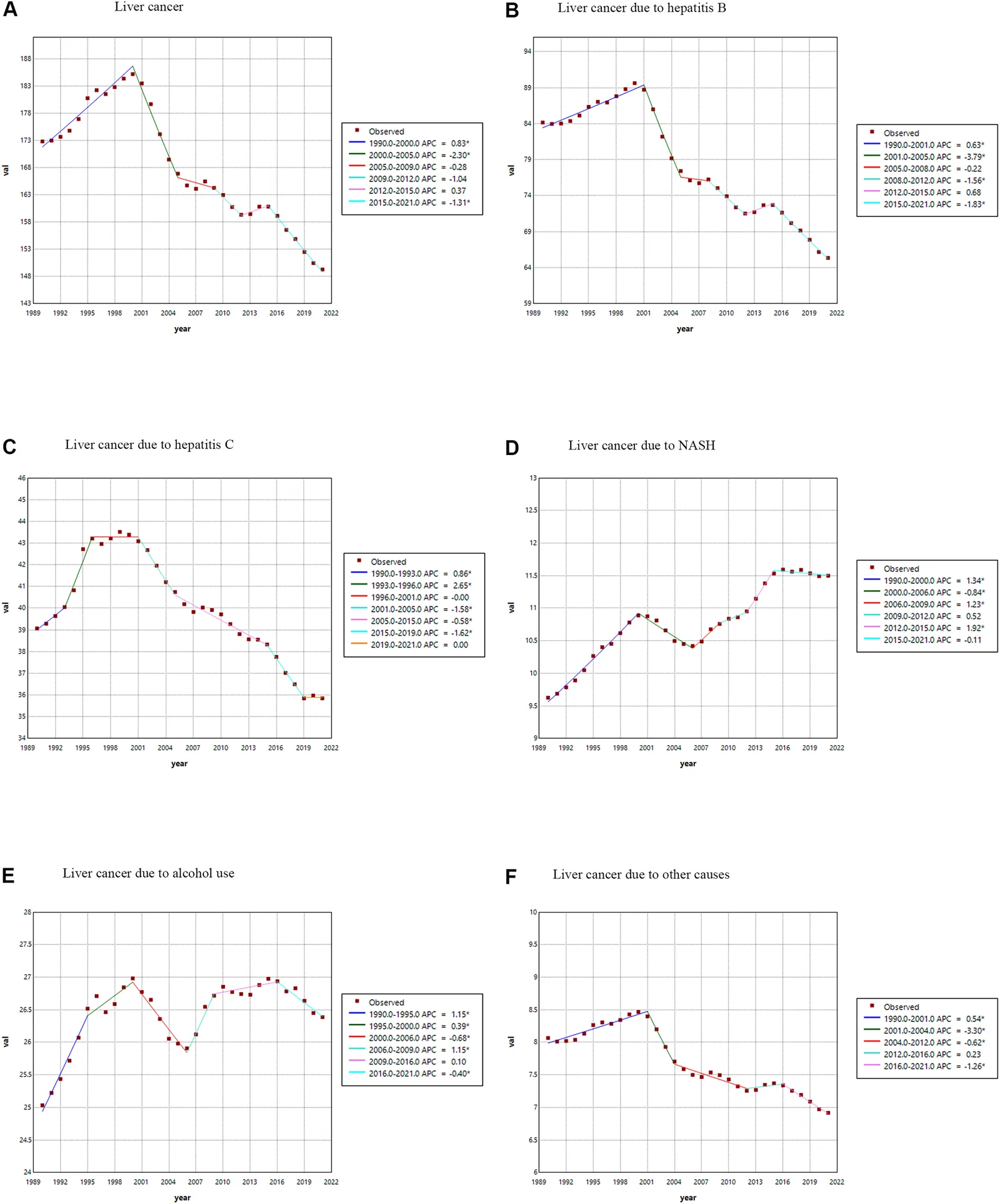
**Joinpoint regression analysis of temporal trends in the age-standardized DALY rate (ASDR) of liver cancer among both sexes globally from 1990 to 2021. (A)** Global liver cancer ASDR exhibited complex nonlinear trends with multiple significant inflection points. **(B)** Hepatitis B-associated ASDR demonstrated a consistent declining trajectory. **(C)** Hepatitis C-related ASDR showed an initial rise followed by stabilization and subsequent decline in recent years. **(D)** NASH-associated ASDR displayed the most substantial increasing trend among all etiological classifications. **(E)** Alcohol-related ASDR remained relatively stable with minor fluctuations but no net change overall. **(F)** Other etiology ASDR maintained general stability with minimal directional changes during the study period. ∗ Indicates that the Annual Percent Change (APC) is significantly different from zero at the alpha = 0.05 level. Abbreviations: DALY, disability-adjusted life year; NASH, non-alcoholic steatohepatitis.

### 3.8 Future forecasts of China and global burden of liver cancer

Longitudinal projections reveal ASDR trajectories between Chinese and global populations through 2035. Globally, overall liver cancer DALY rates exhibited a gradual decline through 2020, with projections indicating stabilization through 2030 within expanding prediction intervals (Fig. 7A). Hepatitis B and C-related rates showed consistent decreasing trajectories throughout the observation period, with these declining trends expected to continue (Fig. 7C and E). In contrast, China demonstrated more pronounced reductions, with overall DALY rates declining substantially from 1990 to 2020 and projected to maintain this downward trajectory through 2030 (Fig. 7B). Specifically, hepatitis B-related rates in China revealed the most rapid decline globally, while hepatitis C-related reductions exceeded global averages (Fig. 7D and F).

**Fig. 7:**
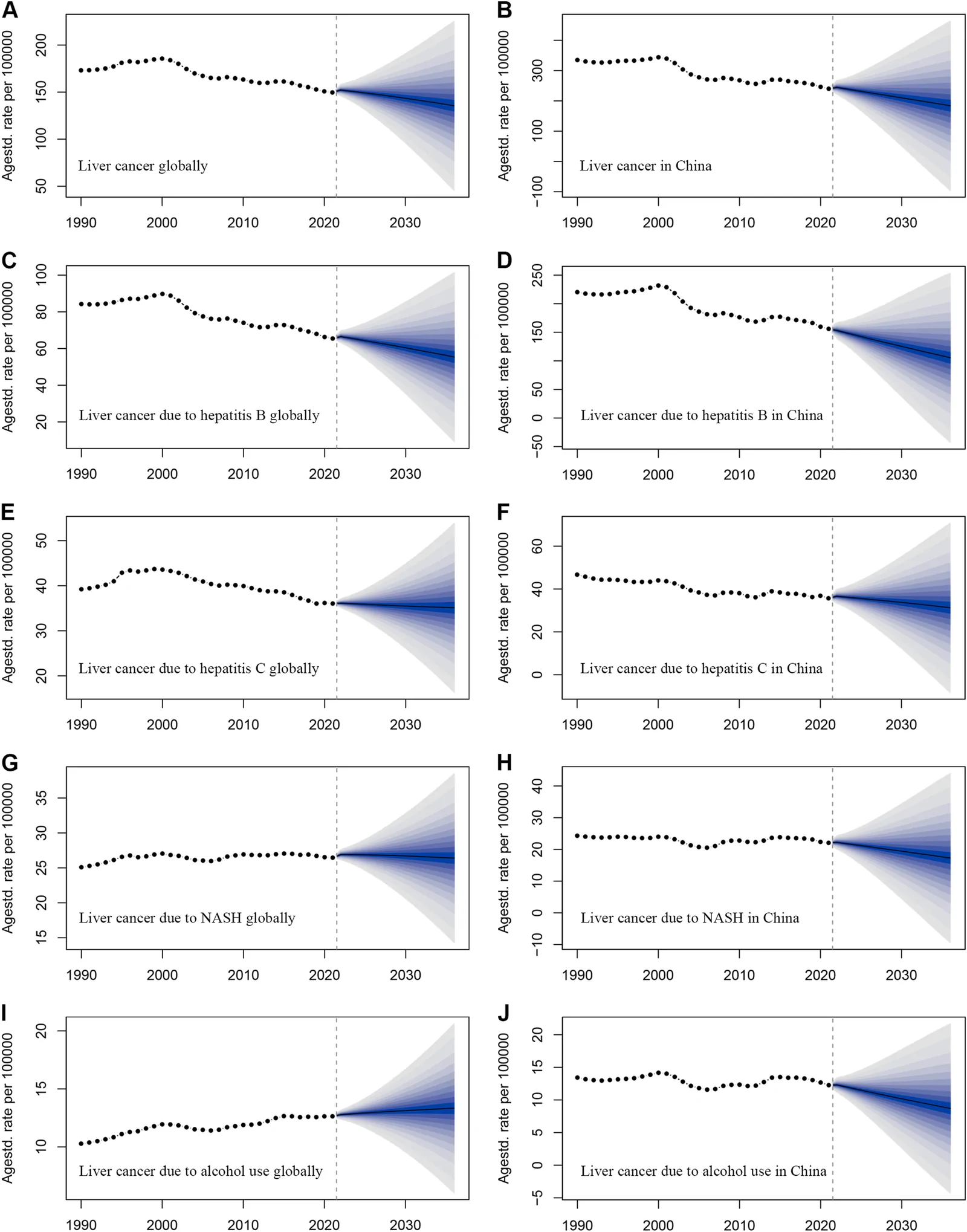
**Future forecasts for the trends in age-standardized DALY rates for liver cancer globally and in China through 2035. (A)** Global liver cancer DALY rates demonstrate a gradual decline from 1990 to 2020, with projected stabilization through 2030 within widening prediction intervals. **(B)** China’s liver cancer DALY rates show a pronounced downward trend from 1990 to 2020, with projections indicating continued decline through 2030. **(C)** Hepatitis B-related liver cancer incidence globally exhibits a steady decrease throughout the observation period, with this declining trend projected to continue. **(D)** China’s hepatitis B-related incidence reveals a dramatic reduction from 1990 to 2020, projected to maintain the world’s most rapid decline through 2030. **(E)** Global hepatitis C-related incidence displays a moderate declining trajectory throughout the study period with persistent reduction expected. **(F)** China’s hepatitis C-related incidence follows a similar declining pattern as the global trend, though with slightly steeper reduction rates. **(G)** NASH-related liver cancer incidence globally shows a concerning upward trend from 1990 to 2020, projected to accelerate through 2030. **(H)** China’s NASH-related incidence reveals the most rapid increase among all etiologies, demonstrating a steep upward trajectory with accelerating growth projected. **(I)** Alcohol-related liver cancer incidence globally maintains relative stability with minor fluctuations, showing no significant trend change through 2030. **(J)** China’s alcohol-related incidence exhibits a modest increasing trend, particularly notable in post-2020 projections despite wider uncertainty intervals.

Notable exceptions to these generally declining patterns were observed in metabolic and alcohol-related etiologies. NASH-related DALY rates exhibited concerning increases globally, with projections suggesting accelerated growth through 2030 (Fig. 7G). China’s NASH-related burden demonstrated the steepest increase among all etiologies, showing a particularly rapid upward trajectory with accelerating growth projected (Fig. 7H). Alcohol-related rates maintained relative stability globally with minimal fluctuations and no significant trend change anticipated (Fig. 7I). However, China’s alcohol-related incidence exhibited a modest but persistent increasing trend, particularly evident in post-2020 projections despite wider uncertainty intervals (Fig. 7J).

## 4 Discussion

The global epidemiological landscape of liver cancer reflects a complex transition shaped by demographic changes and public health interventions.^22^ Our analysis of GBD 2021 data also indicates that while absolute cases of liver cancer and its prevalence, incidence, and DALYs have increased substantially over the past two decades—largely attributable to population growth and aging—age-standardized rates have shown encouraging declines.^6^^,^^23^ Geographical disparities in liver cancer burden reflect distinct stages of epidemiological transition. Regions with historically high viral hepatitis prevalence, including East Asia and sub-Saharan Africa, have demonstrated declining trends, suggesting successful implementation of vaccination programs and antiviral therapies.^24^, ^25^, ^26^ Conversely, high-income regions such as North America and Western Europe exhibit rising burdens despite advanced healthcare systems, indicating the growing influence of metabolic and lifestyle-related risk factors.^27^^,^^28^

Distinct etiological patterns were observed across socioeconomic strata. High-income regions demonstrated a characteristic predominance of metabolic and alcohol-related etiologies, while low- and middle-income regions showed persistent dominance of viral hepatitis-related burden. Our analysis revealed strong positive correlations between regional development levels and proportional contributions of NASH and alcohol-related liver cancer. Conversely, HBV-related burden exhibited an inverse relationship with socioeconomic development. While viral hepatitis infections (HBV and HCV) remain the predominant etiology globally, accounting for approximately 65% of cases, their proportional contribution is decreasing.^29^^,^^30^ Meanwhile, NASH- and alcohol-related liver cancer have shown the most rapid growth since 2000, particularly in high- and middle-SDI countries.^9^^,^^31^ This transition necessitates a re-evaluation of prevention strategies, as the traditional focus on infectious causes must now be complemented with interventions addressing metabolic risk factors and alcohol consumption.^32^

Age-specific patterns reveal particularly concerning trends in older populations, especially in Western countries, with metabolic factors becoming increasingly predominant in this age group.^33^ The global male-to-female disparity shows significant regional variations, with notably lower ratios in low-SDI regions and divergent gender-specific trends across countries.^34^^,^^35^ A distinct age-dependent etiological transition was observed, where NASH-related cases demonstrate a significantly increased proportional representation in advanced age groups, particularly those over 80 years. While prevalence, incidence, and DALYs exhibit highly consistent global distribution patterns, DALYs reveal a disproportionately higher burden in younger cohorts compared to prevalence, reflecting the metric’s sensitivity to premature mortality and years of life lost. These findings identify middle-aged male populations as the primary priority for viral hepatitis screening and treatment. The intersection of these patterns suggests that global control strategies must address a complex nexus of biological susceptibility, regional healthcare access, and sociocultural determinants through age-tailored interventions—prioritizing viral eradication in the workforce and metabolic management in aging populations.

This pattern appears driven by two primary factors: the potential resurgence of viral hepatitis-related cases in certain populations and the continuing rise of metabolic-related liver cancer against a backdrop of slowing population growth.^36^^,^^37^ The convergence of these trends suggests that without intensified intervention efforts, the gains achieved in viral hepatitis control may be offset by the growing burden of metabolic-related liver cancer.

Longitudinal projections through 2035 reveal a concerning U-shaped trajectory: an initial period of decline followed by a projected resurgence in rates across multiple etiological subgroups and regions. The significant declining trajectories of hepatitis B and C-related ASDR reflect the success of expanded vaccination coverage and potent antiviral therapies. In stark contrast, the rising burden of NASH-related liver cancer underscores the intensifying impact of the global obesity and metabolic syndrome epidemic. Meanwhile, the alcohol-related burden remains persistently stable, suggesting that current public health measures have yet to effectively curb mortality from alcohol consumption. China’s marked reduction in hepatitis-related liver cancer burden is largely attributable to its rigorous implementation of infant HBV vaccination since 1992, achieving over 90% coverage, coupled with progressive expansion of antiviral therapy reimbursement under national health insurance.^38^^,^^39^ Concurrently, rapid economic growth and urbanization have driven shifts toward obesogenic lifestyles, characterized by increased consumption of processed foods and reduced physical activity. This nutritional transition, alongside an aging population, has accelerated metabolic syndrome prevalence, thereby elevating NASH-related liver cancer risk.^40^^,^^41^ The coexistence of declining viral hepatitis and rising metabolic etiologies underscores an evolving epidemiological transition, necessitating dual-strategy interventions that sustain vaccination and antiviral programs while integrating public health measures targeting weight management, diabetes prevention, and alcohol consumption. Future policies must address these dual burdens through integrated primary care strategies.^42^ The protective effects of dietary modifications such as Mediterranean-style diets and physical activity promotion offer promising avenues for reducing metabolic-related liver cancer burden.^43^^,^^44^ Future research should focus on understanding the complex interactions between multiple risk factors and developing targeted interventions for high-risk populations.

This study has several limitations inherent to its reliance on the GBD 2021 dataset. The findings are contingent on the accuracy and completeness of the original data sources, which may vary across regions due to differences in diagnostic practices, reporting systems, and healthcare infrastructure.^45^ Methodological constraints include the aggregation of diverse liver cancer subtypes under broader categories, which may obscure important etiological distinctions. Additionally, the analysis could not fully account for contextual factors such as regional variations in healthcare access, screening protocols, or specific policy interventions.^46^ Despite these limitations, the consistent patterns observed across multiple metrics support the robustness of the primary findings. Future refinements to the GBD framework—including more granular subphenotype classification and integration of local health system data—would enhance the precision of burden estimates and better capture national and regional epidemiological nuances.

## 5 Conclusions

The global burden of liver cancer demonstrates a complex epidemiological transition, characterized by declining age-standardized rates amid rising absolute cases—driven by population growth and aging—while revealing significant geographical and etiological heterogeneity. Viral hepatitis (HBV/HCV) remains dominant but is decreasing, while NASH and alcohol-related etiologies surge, especially in high-income regions. Future projections indicate a concerning U-shaped trajectory post-2035, driven by metabolic trends and potential viral resurgence. Addressing this dual burden demands integrated strategies: sustaining viral hepatitis control through vaccination and antiviral therapies, while implementing population-level interventions targeting obesity, alcohol use, and promoting healthy diets and active lifestyles to mitigate emerging metabolic risks.

## Authors’ contributions

**Xue Zhou:** Writing – original draft, Visualization, Validation, Software, Funding acquisition, Formal analysis, Data curation. **Mingle Huang:** Visualization, Validation, Funding acquisition, Data curation. **Xiaofen Li:** Writing – review & editing, Visualization, Validation, Data curation. **Minjie Zhao:** Writing – review & editing, Visualization, Validation, Data curation. **Moushen Tang:** Writing – review & editing, Visualization, Validation, Supervision, Funding acquisition, Data curation.

## AI declaration

DeepSeek (DeepSeek V4, api.deepseek.com) was used solely to improve grammar and readability. All scientific content, interpretation, and final approval were performed by the authors.

## Data availability statement

The datasets analyzed in this study are accessible through the GHDx query tool. (https://vizhub.healthdata.org/gbd-results/). The datasets used and/or analyzed during the study are available from the corresponding authors upon reasonable request.

## Declaration of competing interest

The authors declare that there is no conflicts of interest.
