# Supplementary material for: The changing etiology of liver cancer: A comparative study of global and Chinese burden trends from hepatitis dominance to metabolic risks (1990–2021) and forecasts to 2035
Source: Liver Res. 2026 Jul 29;10(3):231–44. doi: 10.1016/j.livres.2026.07.001 (PMC13649511; doi:10.1016/j.livres.2026.07.001)
Supplement: Supplementary file 1 [file mmc1.docx]

**Supplementary materials**

**Supplemental Fig.1 NASH‑related liver cancer incidence in relation to socio-demographic development.** Age-standardized incidence rates (ASIR) of NASH‑related liver cancer (per 100,000 population) are shown in relation to the 2019 Socio-demographic Index (SDI) for the global population and 21 GBD regions from 1990 to 2021. Points represent region-specific estimates over time. The solid line indicates the smoothed global association, with shaded areas denoting 95% uncertainty intervals.

**Supplemental Fig.2 Association between Sociodemographic Index (SDI) and age-standardized DALY rates due to NASH-related liver cancer.** Age-standardized rates of DALYs (ASDR) attributable to NASH-related liver cancer (per 100,000 population) are shown in relation to the 2019 Sociodemographic Index (SDI) across the global population and 21 GBD regions from 1990 to 2021. Each point represents region-specific estimates across time. The solid curve depicts the smoothed global association between SDI and ASDR, while the shaded band indicates the 95% uncertainty interval.
