# Supplementary material for: The changing etiology of liver cancer: A comparative study of global and Chinese burden trends from hepatitis dominance to metabolic risks (1990–2021) and forecasts to 2035
Source: Liver Res. 2026 Jul 29;10(3):231–44. doi: 10.1016/j.livres.2026.07.001 (PMC13649511; doi:10.1016/j.livres.2026.07.001)
Supplement: Supplementary file 2 [file mmc2.docx]

**Supplementary materials**

**Supplemental Table 1**

Global age-standardized prevalence of liver cancer in 2021.

| Location | 2021 ASPR (1/100,000) R (95% CI) |
| --- | --- |
| Kazakhstan | 5.080159 (4.207244-5.8974) |
| Taiwan, China | 17.08121 (15.30765-19.14383) |
| Kiribati | 6.5096 (4.69508-9.108213) |
| Fiji | 5.053775 (3.485618-7.005626) |
| Cambodia | 7.174614 (4.005363-12.52717) |
| Georgia | 4.232395 (3.691689-4.841721) |
| Democratic People's Republic of Korea | 8.545206 (5.563985-12.1048) |
| Azerbaijan | 8.643434 (4.989288-14.34228) |
| Armenia | 6.672311 (5.511713-7.876601) |
| Viet Nam | 15.0979 (10.93257-21.29517) |
| China | 13.28745 (10.74703-16.41414) |
| Thailand | 13.44862 (10.15809-17.94713) |
| Timor-Leste | 3.128129 (1.99158-5.037232) |
| Philippines | 6.780584 (5.552313-8.136913) |
| Vanuatu | 4.443822 (2.924673-7.051187) |
| Sri Lanka | 1.617249 (1.038788-2.286358) |
| Tonga | 23.2513 (16.29775-32.43442) |
| Micronesia (Federated States of) | 5.411528 (3.484731-8.571301) |
| Mongolia | 76.56917 (58.82205-97.89076) |
| Maldives | 4.825162 (3.564608-6.288594) |
| Uzbekistan | 4.658732 (3.423685-6.007355) |
| Myanmar | 3.744341 (1.912389-7.147224) |
| Malaysia | 6.413133 (5.084539-7.955304) |
| Kyrgyzstan | 3.574855 (2.911226-4.37666) |
| Tajikistan | 4.618318 (2.893162-6.945686) |
| Papua New Guinea | 2.759231 (1.32277-6.722999) |
| Solomon Islands | 5.093887 (3.314162-8.222238) |
| Samoa | 5.213251 (3.854854-6.871002) |
| Lao People's Democratic Republic | 7.376936 (5.038067-10.07929) |
| Turkmenistan | 5.842079 (4.437082-7.725437) |
| Marshall Islands | 4.102081 (2.438614-7.009966) |
| Czechia | 2.745639 (2.197147-3.334654) |
| Republic of Moldova | 4.048904 (3.470052-4.753716) |
| Hungary | 1.927545 (1.539382-2.368883) |
| Indonesia | 4.361824 (2.541844-6.809545) |
| Bulgaria | 3.684343 (2.739142-4.945887) |
| Lithuania | 4.607924 (3.948637-5.376883) |
| Croatia | 3.764255 (3.004916-4.746199) |
| Bosnia and Herzegovina | 6.046677 (4.509475-7.553868) |
| Latvia | 3.79084 (3.167878-4.479001) |
| Andorra | 15.22179 (10.45262-20.90695) |
| Estonia | 4.785546 (3.99318-5.758184) |
| Albania | 8.947637 (5.991139-13.32773) |
| Belarus | 3.637044 (2.861705-4.571486) |
| New Zealand | 10.23417 (9.070758-11.43654) |
| Slovenia | 5.320027 (4.416533-6.50632) |
| Australia | 9.579008 (8.498276-10.73801) |
| Singapore | 13.67541 (12.16308-15.27828) |
| Slovakia | 4.225347 (3.09865-5.796276) |
| Brunei Darussalam | 11.22612 (8.747513-14.48954) |
| Serbia | 5.356992 (3.939166-7.029673) |
| North Macedonia | 8.089035 (6.188233-10.35225) |
| Japan | 20.10438 (17.94339-21.64818) |
| Republic of Korea | 38.16453 (31.5098-46.61197) |
| Romania | 3.254513 (2.703567-3.93182) |
| Montenegro | 6.912901 (5.037616-9.258937) |
| Denmark | 4.864015 (4.281254-5.545487) |
| Cyprus | 4.308497 (3.236412-5.795807) |
| Russian Federation | 3.207019 (2.974864-3.457047) |
| Finland | 10.49708 (9.183029-11.70401) |
| Luxembourg | 5.887154 (5.242442-6.598223) |
| Belgium | 5.740226 (5.133546-6.429192) |
| Austria | 9.489321 (8.373507-10.57434) |
| Ireland | 4.984832 (4.309803-5.586518) |
| Ukraine | 1.795684 (1.349648-2.24701) |
| Chile | 3.485382 (3.125185-3.858584) |
| Belize | 2.460653 (2.151028-2.784987) |
| Poland | 1.835149 (1.662579-2.005537) |
| Brazil | 2.042421 (1.916915-2.155377) |
| Mauritius | 0.801963 (0.738122-0.85389) |
| Uruguay | 2.592343 (2.220024-2.955736) |
| Equatorial Guinea | 6.079046 (3.701138-9.643754) |
| Nicaragua | 3.28903 (2.50161-4.372295) |
| Oman | 4.117855 (3.065614-5.295012) |
| Bahamas | 3.170944 (2.539575-3.956186) |
| Panama | 3.566729 (2.871521-4.332241) |
| Italy | 8.58795 (7.883323-9.267753) |
| Saint Vincent and the Grenadines | 2.850915 (2.486002-3.27714) |
| Trinidad and Tobago | 2.28845 (1.739667-2.904162) |
| Malawi | 6.12451 (4.356519-8.814206) |
| Iceland | 6.546973 (5.706282-7.499262) |
| Germany | 9.070647 (8.198609-10.0645) |
| Egypt | 19.27928 (15.03732-24.02445) |
| Tunisia | 2.280126 (1.566553-3.186778) |
| Turkiye | 3.178869 (2.491574-4.0296) |
| Iran (Islamic Republic of) | 3.208797 (2.801129-3.690728) |
| Honduras | 3.658442 (2.677338-5.036297) |
| El Salvador | 1.792443 (1.41609-2.243929) |
| Jordan | 1.68367 (1.227491-2.250843) |
| Bahrain | 5.06123 (3.907033-6.518448) |
| France | 11.40672 (9.985388-12.92415) |
| Guyana | 2.363991 (1.787677-3.049664) |
| Grenada | 3.796595 (3.273431-4.323014) |
| Norway | 6.812897 (6.320238-7.303604) |
| Kenya | 4.637734 (3.580874-6.130767) |
| Haiti | 1.57733 (0.857592-2.887954) |
| Uganda | 8.514605 (6.190898-11.46623) |
| Guatemala | 5.438813 (4.641347-6.325508) |
| Somalia | 9.080559 (4.638153-16.56606) |
| Ecuador | 2.8371 (2.25052-3.56378) |
| United Republic of Tanzania | 5.448179 (3.827867-7.892402) |
| India | 3.167485 (2.787542-3.565433) |
| Benin | 14.09283 (10.42072-18.53051) |
| Yemen | 2.422156 (1.424267-4.163576) |
| Dominican Republic | 2.082016 (1.516088-2.760385) |
| Dominica | 3.542275 (2.69247-4.618464) |
| American Samoa | 7.016813 (5.444918-8.99564) |
| Burundi | 3.106874 (1.972964-5.220817) |
| Canada | 8.121803 (7.351875-9.016656) |
| Bhutan | 4.681714 (2.971145-6.668485) |
| Syrian Arab Republic | 5.604331 (3.892156-7.515965) |
| United States of America | 9.313234 (8.85279-9.645335) |
| Switzerland | 7.315875 (6.507125-8.256863) |
| Togo | 8.411187 (5.589243-13.05711) |
| Zimbabwe | 15.25687 (9.727832-21.66893) |
| Seychelles | 4.593411 (3.681688-5.687679) |
| Colombia | 2.367918 (1.94713-2.842352) |
| Sao Tome and Principe | 2.558692 (1.63321-4.363136) |
| Libya | 9.180693 (6.510505-12.52262) |
| Sweden | 3.517687 (3.07231-4.064385) |
| Venezuela (Bolivarian Republic of) | 3.005922 (2.301329-3.828844) |
| Antigua and Barbuda | 2.709633 (2.523861-2.907504) |
| Zambia | 5.620265 (2.617853-12.73624) |
| Comoros | 5.27432 (3.581982-7.56878) |
| Guinea | 22.14854 (14.53772-32.02383) |
| Mali | 31.33368 (23.58484-40.67203) |
| Greenland | 6.575055 (4.927501-8.681143) |
| Bermuda | 1.89568 (1.590478-2.318393) |
| Israel | 3.173418 (2.817506-3.531696) |
| United Arab Emirates | 10.87107 (8.07214-14.46152) |
| Qatar | 15.76815 (11.69766-21.46724) |
| Nigeria | 5.015155 (3.512974-7.009051) |
| Cook Islands | 12.84915 (9.692066-17.31757) |
| Djibouti | 4.941552 (3.201979-7.451647) |
| Congo | 7.795949 (3.947708-14.82769) |
| Peru | 2.569406 (1.797969-3.441896) |
| Guam | 6.974647 (5.781407-8.099189) |
| Monaco | 11.62516 (8.234088-15.89187) |
| Cabo Verde | 13.37852 (9.092585-17.96501) |
| Costa Rica | 6.232264 (5.371882-7.16109) |
| Morocco | 0.594487 (0.425656-0.766898) |
| Central African Republic | 7.904732 (3.602789-15.37392) |
| Nepal | 3.403559 (2.208004-4.775357) |
| Botswana | 6.171007 (3.43252-12.19316) |
| Mauritania | 20.90214 (11.12729-30.3027) |
| Mozambique | 22.04157 (12.41154-41.10598) |
| Eritrea | 4.244494 (2.735627-7.076446) |
| Cuba | 1.69972 (1.451714-1.963124) |
| Eswatini | 21.29194 (8.397235-44.99057) |
| Rwanda | 4.764791 (3.295467-6.907829) |
| Barbados | 2.529225 (1.987254-3.231082) |
| Northern Mariana Islands | 7.373929 (6.018761-8.996911) |
| South Sudan | 7.906267 (4.848128-11.56965) |
| Democratic Republic of the Congo | 3.839807 (1.919945-8.131985) |
| Afghanistan | 7.122622 (4.930319-9.86237) |
| Mexico | 3.4076 (2.991424-3.821683) |
| Portugal | 6.024474 (5.402507-6.780957) |
| Spain | 9.384911 (8.203013-10.59764) |
| South Africa | 6.935143 (5.994004-8.013435) |
| Palau | 8.752994 (6.340258-11.68856) |
| Bangladesh | 2.565017 (1.905442-3.405216) |
| Kuwait | 1.359586 (1.097109-1.629636) |
| C么te d'Ivoire | 4.71825 (3.026578-6.864816) |
| Angola | 9.198718 (2.437384-22.364) |
| Sudan | 4.097648 (2.710653-5.757049) |
| Saint Lucia | 1.673054 (1.359568-2.033134) |
| Lesotho | 16.77767 (7.310008-37.20337) |
| Chad | 14.62624 (8.936231-23.54147) |
| Suriname | 2.79912 (2.048582-3.789669) |
| Puerto Rico | 3.50018 (2.889203-4.163263) |
| Netherlands | 2.800973 (2.546765-3.070827) |
| Argentina | 1.257675 (1.129043-1.385873) |
| Guinea-Bissau | 20.26843 (13.81244-27.37213) |
| Niue | 5.673971 (3.831817-8.534481) |
| Senegal | 9.868191 (7.116744-13.28119) |
| Niger | 9.84921 (6.489161-15.69567) |
| Burkina Faso | 19.87523 (9.945472-33.04524) |
| Ghana | 8.569033 (5.849154-12.44366) |
| Tokelau | 5.386692 (3.415691-8.362498) |
| Gabon | 10.63416 (6.560756-16.22125) |
| Tuvalu | 5.113021 (3.48312-7.399028) |
| Gambia | 37.09478 (24.44514-54.50432) |
| Saint Kitts and Nevis | 3.340307 (2.750368-3.93567) |
| Cameroon | 13.30128 (7.516441-22.19011) |
| Malta | 4.02455 (3.421593-4.720577) |
| Paraguay | 3.472132 (2.475715-4.664731) |
| Greece | 6.514441 (6.042194-7.008428) |
| Bolivia (Plurinational State of) | 3.581454 (2.444755-5.11486) |
| United States Virgin Islands | 2.630972 (1.812422-3.830227) |
| Madagascar | 3.089383 (2.020305-4.718442) |
| San Marino | 3.689073 (2.332785-5.420949) |
| Pakistan | 4.720567 (3.578963-6.100704) |
| Nauru | 6.509074 (4.098208-9.743558) |
| Iraq | 5.065688 (3.673965-6.658667) |
| Jamaica | 1.688521 (1.288238-2.172568) |
| Lebanon | 2.749036 (2.121101-3.478182) |
| United Kingdom | 11.35953 (10.81571-11.7876) |
| Palestine | 5.966905 (4.747443-7.518507) |
| Namibia | 3.59893 (2.595925-4.895786) |
| Algeria | 2.153715 (1.613735-2.807755) |
| Liberia | 18.92648 (12.3822-26.80162) |
| Sierra Leone | 10.52131 (7.319174-14.62923) |
| Saudi Arabia | 6.324436 (4.680338-8.293216) |
| Ethiopia | 2.890108 (1.952626-4.650525) |
| Central Asia | 7.379637 (6.26293-8.616156) |
| Eastern Europe | 2.981406 (2.769025-3.197811) |
| Central Europe | 3.214224 (2.878265-3.58674) |
| Global | 8.680531 (7.90453-9.673047) |
| High-income Asia Pacific | 25.26005 (22.43141-27.91537) |
| Western Europe | 8.884447 (8.322917-9.382658) |
| Central Latin America | 3.245111 (2.887785-3.630249) |
| High-income North America | 9.188647 (8.720622-9.528779) |
| Australasia | 9.676507 (8.684066-10.75707) |
| Southern Latin America | 1.995285 (1.798231-2.197117) |
| Southern Sub-Saharan Africa | 8.234974 (6.958318-9.621726) |
| Andean Latin America | 2.80706 (2.180043-3.536926) |
| Low-middle SDI | 4.736397 (4.228616-5.308134) |
| Low SDI | 6.047601 (4.927169-7.737142) |
| High-middle SDI | 8.423713 (7.205929-9.96201) |
| Caribbean | 2.083675 (1.792105-2.413668) |
| High SDI | 12.20587 (11.30382-12.85695) |
| Middle SDI | 8.420739 (7.258784-9.987097) |
| Tropical Latin America | 2.078113 (1.945093-2.199836) |
| North Africa and Middle East | 5.740013 (4.902378-6.633177) |
| Central Sub-Saharan Africa | 5.524138 (2.593578-11.27001) |
| Eastern Sub-Saharan Africa | 5.995561 (4.468394-7.912447) |
| South Asia | 3.316122 (2.908373-3.717651) |
| Western Sub-Saharan Africa | 9.85398 (8.053662-11.65824) |
| Western Africa | 9.313221 (7.647899-11.10404) |
| Northern Africa | 9.340691 (7.408175-11.40784) |
| Eastern Africa | 4.722838 (3.694337-6.220177) |
| Sub-Saharan Africa - WB | 7.649044 (6.288996-9.5159) |
| South Asia - WB | 3.325729 (2.908071-3.732074) |
| Middle East & North Africa - WB | 6.310619 (5.324984-7.370839) |
| Commonwealth Middle Income | 3.810349 (3.370693-4.212609) |
| Latin America & Caribbean - WB | 2.555663 (2.340826-2.785617) |
| Europe & Central Asia - WB | 6.283619 (5.905113-6.611488) |
| Commonwealth High Income | 10.04075 (9.47152-10.48071) |
| Western Pacific Region | 15.17078 (13.03495-17.71276) |
| East Asia & Pacific - WB | 13.96812 (12.13255-16.29058) |
| Eastern Mediterranean Region | 6.344076 (5.444203-7.384815) |
| South-East Asia Region | 3.933747 (3.469875-4.535891) |
| European Region | 6.260586 (5.884383-6.588315) |
| Africa | 8.058251 (6.78276-9.532845) |
| Commonwealth Low Income | 4.892773 (3.638643-6.561788) |
| Asia | 10.11773 (8.949277-11.54866) |
| Region of the Americas | 5.4839 (5.190467-5.712141) |
| African Region | 7.404712 (6.119571-9.144226) |
| America | 5.4839 (5.190467-5.712141) |
| Europe | 6.357127 (5.967711-6.68845) |
| Central Africa | 7.020923 (4.944511-10.37637) |
| Basic Health System | 9.583631 (8.171283-11.47424) |
| Southern Africa | 9.591379 (7.452489-12.28741) |
| North America | 9.18761 (8.719718-9.527885) |
| Advanced Health System | 10.1208 (9.443953-10.6274) |
| Limited Health System | 3.990163 (3.494799-4.529399) |
| Minimal Health System | 11.75367 (9.251484-15.3186) |
| Southeast Asia | 7.654937 (6.025008-9.844202) |
| Oceania | 3.72319 (2.474877-6.684246) |
| East Asia | 13.28394 (10.79003-16.31501) |

Abbreviation: CI, confidence interval.

**Supplemental Table 2**

Global age-standardized incidence of liver cancer in 2021.

| Location | 2021 ASIR (1/100,000) R (95% CI) |
| --- | --- |
| Viet Nam | 12.23098543 (8.837875375-17.16708225) |
| Fiji | 4.531642216 (3.144562728-6.222653651) |
| Bulgaria | 3.328881972 (2.475259033-4.434609879) |
| Kazakhstan | 4.695162016 (3.899919425-5.479259137) |
| Tonga | 20.29043446 (14.3273126-27.96408067) |
| Mongolia | 74.56224662 (57.15628531-94.46409293) |
| China | 9.517868041 (7.719586824-11.78390433) |
| Malaysia | 5.585754606 (4.436764094-6.941580062) |
| Myanmar | 3.245437143 (1.687374122-6.202050531) |
| Taiwan, China | 11.20614767 (9.9764434-12.62402114) |
| Armenia | 6.175836417 (5.094239328-7.328691662) |
| Turkmenistan | 5.23133867 (3.934666516-6.902357318) |
| Marshall Islands | 3.711823624 (2.232799362-6.265521577) |
| Sri Lanka | 1.369285243 (0.876665292-1.918112566) |
| Cambodia | 6.529531399 (3.664755955-11.21882429) |
| Papua New Guinea | 2.403377583 (1.137874322-5.919666479) |
| Solomon Islands | 4.432385853 (2.931042116-7.247700488) |
| Lao People's Democratic Republic | 6.583430638 (4.511214989-8.929106613) |
| Timor-Leste | 2.778282257 (1.787807459-4.455242192) |
| Bosnia and Herzegovina | 5.698239732 (4.248538859-7.168778661) |
| Georgia | 3.910565995 (3.397467317-4.464886109) |
| Kyrgyzstan | 3.345277413 (2.739992596-4.079144447) |
| Croatia | 3.04782671 (2.440464722-3.844785529) |
| Vanuatu | 4.137487537 (2.735757057-6.500096047) |
| Maldives | 4.346500345 (3.165746816-5.726254923) |
| Romania | 2.897839346 (2.415290607-3.483144314) |
| Czechia | 2.469112926 (1.99296518-2.986672057) |
| Albania | 7.604126019 (5.097361156-11.02220902) |
| Tajikistan | 4.267144495 (2.695487414-6.324111602) |
| Democratic People's Republic of Korea | 6.924288756 (4.638174546-9.786550543) |
| Kiribati | 5.828522252 (4.229323099-7.970377729) |
| Indonesia | 3.89715491 (2.216139444-6.061269213) |
| Thailand | 10.71785684 (8.030198531-14.31512361) |
| Micronesia (Federated States of) | 4.856954905 (3.112614485-7.580384266) |
| Republic of Korea | 19.9383234 (16.27440433-24.48645643) |
| North Macedonia | 8.030064511 (6.171330068-10.25515062) |
| Philippines | 5.917809223 (4.859657152-7.108721965) |
| Cuba | 1.465309412 (1.260863934-1.69203571) |
| Samoa | 4.583896594 (3.376879491-6.016741555) |
| Azerbaijan | 8.02149529 (4.570428385-13.16545355) |
| Suriname | 2.508767317 (1.830443157-3.377200448) |
| Uzbekistan | 4.185706755 (3.085803969-5.411767219) |
| Cyprus | 3.329100599 (2.525021148-4.480600108) |
| Argentina | 1.100096769 (0.987984386-1.210977678) |
| Poland | 1.681467389 (1.520477863-1.841002611) |
| Guyana | 2.163046314 (1.649971139-2.762123464) |
| Germany | 4.958472158 (4.460011807-5.494406642) |
| Italy | 5.1512546 (4.714974034-5.527759192) |
| Malta | 2.926784767 (2.508894104-3.42232193) |
| Norway | 3.956877693 (3.643102938-4.20433517) |
| Belize | 2.216238757 (1.945447574-2.5048213) |
| Serbia | 4.942439675 (3.657727654-6.44000901) |
| Hungary | 1.690548998 (1.367501318-2.06373591) |
| Singapore | 7.355551333 (6.557394463-8.08660694) |
| Australia | 5.889282717 (5.250520338-6.61561089) |
| Belgium | 4.163066699 (3.736617133-4.652599383) |
| Morocco | 0.511681471 (0.362046526-0.649124021) |
| Belarus | 2.701569417 (2.135708328-3.371252654) |
| Bahamas | 2.853875621 (2.30991896-3.518470822) |
| Canada | 5.016441845 (4.500898435-5.55781699) |
| Luxembourg | 4.251991151 (3.79397429-4.736203447) |
| Israel | 2.395532543 (2.124033782-2.657208067) |
| El Salvador | 1.589779788 (1.248906497-1.993565012) |
| Paraguay | 3.001496724 (2.126209854-3.986142085) |
| Slovenia | 4.500373335 (3.737118024-5.507400866) |
| Grenada | 3.693840211 (3.177905958-4.178862515) |
| Brunei Darussalam | 9.805897812 (7.66696422-12.65105208) |
| Chile | 3.112434761 (2.7820422-3.460828077) |
| Oman | 3.523515183 (2.596575108-4.555069987) |
| Austria | 5.844431488 (5.18671511-6.471434768) |
| Denmark | 3.22738922 (2.860929873-3.676117795) |
| Colombia | 2.067911736 (1.729375149-2.428994417) |
| Ireland | 3.47374454 (2.982755269-3.915707389) |
| Haiti | 1.431462655 (0.765714723-2.717213616) |
| France | 6.909176057 (6.01916598-7.762958075) |
| Palestine | 5.610026253 (4.464335564-7.087006054) |
| Angola | 8.316013025 (2.14428928-20.74552495) |
| Republic of Moldova | 2.841041481 (2.488017588-3.21953188) |
| Lithuania | 3.47860923 (3.002289158-4.021140561) |
| Gabon | 9.938898594 (6.166701087-15.2160616) |
| Costa Rica | 5.568456471 (4.775651263-6.419615523) |
| Finland | 5.456236357 (4.834349732-6.079951264) |
| Saint Lucia | 1.482395131 (1.207656827-1.785656494) |
| Trinidad and Tobago | 2.044437266 (1.551121221-2.567783962) |
| Jamaica | 1.530239492 (1.172874629-1.955475146) |
| New Zealand | 5.370177794 (4.830854249-5.937086356) |
| Sweden | 2.762052876 (2.380385965-3.166324702) |
| Bolivia (Plurinational State of) | 3.28727772 (2.164107962-4.755886251) |
| Iran (Islamic Republic of) | 2.702967543 (2.392385633-3.036044121) |
| Dominica | 3.386587715 (2.604272377-4.419526975) |
| Brazil | 1.774175369 (1.662562918-1.867120481) |
| Jordan | 1.445281532 (1.051494491-1.937269165) |
| Antigua and Barbuda | 2.531617608 (2.362578096-2.714901405) |
| Montenegro | 6.270911513 (4.598717056-8.378067407) |
| Netherlands | 2.421987486 (2.19461434-2.658946169) |
| Iceland | 4.47648863 (3.930169376-5.086910572) |
| Eswatini | 19.71709572 (7.945157257-40.5562364) |
| Bahrain | 4.824902908 (3.733588083-6.096309384) |
| Slovakia | 3.697583904 (2.685517574-5.08656432) |
| Barbados | 2.357239159 (1.883424183-2.960976399) |
| Afghanistan | 6.243843329 (4.342273767-9.000704989) |
| Nepal | 2.981546481 (1.91072511-4.19876464) |
| Panama | 3.070588091 (2.44376604-3.735804039) |
| Malawi | 4.895893489 (3.693430418-6.427969156) |
| Eritrea | 3.776818579 (2.517867118-5.964331913) |
| Gambia | 32.30770748 (20.63646209-47.9480167) |
| Seychelles | 4.077670014 (3.284973851-5.060873522) |
| Dominican Republic | 1.820910313 (1.338414299-2.411634861) |
| Ecuador | 2.629917844 (2.078909217-3.305152344) |
| Niger | 8.937520429 (5.977012462-14.49002853) |
| Guinea | 19.11376409 (12.21516724-28.31611545) |
| Burkina Faso | 17.21563095 (8.822142554-29.63864909) |
| Mexico | 3.030990367 (2.683273455-3.383921828) |
| Saint Vincent and the Grenadines | 2.602762262 (2.277495543-2.959762178) |
| Libya | 7.368553732 (5.349118474-10.04550161) |
| Qatar | 14.42418719 (10.59320538-19.49924123) |
| Tunisia | 1.915793637 (1.282715648-2.674281209) |
| Cabo Verde | 12.38802915 (8.086182742-16.66046747) |
| Sao Tome and Principe | 2.291934174 (1.365435782-3.81606999) |
| Liberia | 17.05162651 (11.25002657-23.59506932) |
| American Samoa | 6.212282621 (4.876763708-7.945414099) |
| Sierra Leone | 9.165575169 (6.452918302-12.79720488) |
| Spain | 5.93809326 (5.167756302-6.691910458) |
| Botswana | 5.702400527 (3.204140559-10.91806522) |
| Mauritania | 19.12776973 (10.17542012-27.93186223) |
| Guatemala | 4.990252377 (4.271572849-5.757665077) |
| C么te d'Ivoire | 3.863364767 (2.272996164-5.706502704) |
| Ghana | 7.762061191 (5.062717747-11.26518103) |
| Zambia | 4.813481616 (2.266975319-10.51137927) |
| Kenya | 4.137966219 (3.208649959-5.452666731) |
| Nigeria | 4.552760039 (3.216311269-6.383712331) |
| Cameroon | 11.90076464 (6.657256275-19.71481198) |
| Chad | 13.1278708 (8.07197308-21.70124751) |
| Bhutan | 3.817440833 (2.424558847-5.367594495) |
| Senegal | 9.011391777 (6.505009679-12.28400447) |
| Mali | 27.43658367 (20.50967826-35.94300377) |
| United Republic of Tanzania | 4.579095497 (3.35997791-6.391231462) |
| Guinea-Bissau | 18.6698542 (12.87527443-24.54281297) |
| Iraq | 4.612059368 (3.270102065-6.042233137) |
| Congo | 7.277041844 (3.712443671-13.84985682) |
| Togo | 7.76136404 (5.095924019-12.02312823) |
| Ukraine | 1.366967497 (0.995246763-1.749717551) |
| Kuwait | 1.141158814 (0.92165406-1.369037876) |
| Russian Federation | 2.566934161 (2.36153389-2.78035076) |
| Saudi Arabia | 5.975904011 (4.390828144-7.806394543) |
| Egypt | 18.36598345 (14.37137187-22.84965879) |
| Benin | 12.33127522 (9.270653278-16.26029724) |
| Lesotho | 15.91128895 (7.2316937-34.06407527) |
| Latvia | 3.103878613 (2.585216696-3.644410859) |
| Portugal | 4.819213854 (4.340047388-5.323213212) |
| Uganda | 7.082990992 (5.125192148-9.745069214) |
| Djibouti | 4.425662099 (2.96174729-6.42801613) |
| United Kingdom | 6.415710121 (6.031144026-6.666511004) |
| Yemen | 2.138185486 (1.247912275-3.692159028) |
| United States of America | 5.577503522 (5.202172314-5.793873765) |
| Ethiopia | 2.522341714 (1.730047481-3.978259212) |
| Estonia | 3.727887027 (3.126289383-4.422327047) |
| Uruguay | 2.314332996 (1.984109223-2.639112174) |
| Palau | 7.240602476 (5.271501786-9.739838466) |
| Bangladesh | 2.151177835 (1.592159581-2.828944) |
| India | 2.733408269 (2.425449806-3.066468814) |
| South Africa | 6.356713132 (5.522476454-7.356427849) |
| Algeria | 1.773579286 (1.327776206-2.322102077) |
| Rwanda | 4.272948497 (2.923025674-5.969332929) |
| Tuvalu | 4.555390486 (3.145568779-6.582899079) |
| Madagascar | 2.722920736 (1.818969804-4.197733324) |
| Equatorial Guinea | 5.622260814 (3.445160691-8.790633449) |
| Namibia | 3.140511988 (2.332942972-4.20341958) |
| Pakistan | 3.716827109 (2.789829187-4.804836692) |
| Zimbabwe | 14.63073843 (9.55251203-20.37235421) |
| Saint Kitts and Nevis | 3.202097161 (2.641937958-3.699912986) |
| Switzerland | 4.390125513 (3.904109438-4.890631663) |
| Syrian Arab Republic | 4.978210039 (3.557938926-6.630324122) |
| Comoros | 4.657904086 (3.087272873-6.623277646) |
| Lebanon | 2.205535758 (1.705422763-2.761514682) |
| Tokelau | 4.640648335 (2.972550081-7.153603548) |
| Somalia | 8.513705138 (4.438761048-15.23619875) |
| South Sudan | 6.466081597 (4.195647496-9.399935219) |
| Peru | 2.264334838 (1.578907975-3.073383505) |
| Central African Republic | 7.272511671 (3.242084746-14.4773583) |
| Honduras | 3.618627415 (2.653776555-5.012894857) |
| United States Virgin Islands | 2.426033794 (1.663243787-3.505327099) |
| Cook Islands | 10.66824728 (8.15961653-14.17694559) |
| Turkiye | 2.755881811 (2.144106797-3.4949031) |
| Burundi | 2.774059605 (1.779920834-4.506975288) |
| Guam | 5.601331253 (4.669063571-6.508585309) |
| Niue | 4.858766483 (3.299612723-7.200248701) |
| Japan | 9.892275464 (8.837269137-10.56835085) |
| Nicaragua | 2.921499585 (2.20554234-3.837504362) |
| Andorra | 10.87464295 (7.610509262-14.85800982) |
| Sudan | 3.41897939 (2.296507238-4.866079688) |
| Bermuda | 1.507470015 (1.272973752-1.82731272) |
| United Arab Emirates | 10.51977828 (7.763871993-13.93160987) |
| Greece | 4.396450665 (4.078230588-4.693703605) |
| San Marino | 2.482881447 (1.55815581-3.677177855) |
| Nauru | 5.351970799 (3.450646048-7.988331857) |
| Mozambique | 22.63544204 (12.94551853-42.37060442) |
| Northern Mariana Islands | 6.515437861 (5.325223017-8.021359728) |
| Greenland | 5.903034991 (4.342958705-7.93383871) |
| Venezuela (Bolivarian Republic of) | 2.435719098 (1.922422948-3.084172975) |
| Monaco | 8.27249244 (5.961336921-11.36016382) |
| Puerto Rico | 2.885918263 (2.363828182-3.423515966) |
| Mauritius | 0.695438591 (0.642027641-0.739901871) |
| Democratic Republic of the Congo | 3.610182975 (1.778994836-7.909381407) |
| Global | 6.152430398 (5.578764182-6.896921045) |
| East Asia | 9.511045117 (7.756207671-11.68377837) |
| Eastern Europe | 2.340478409 (2.169677758-2.522159929) |
| Central Europe | 2.869339849 (2.580971472-3.192546708) |
| Southeast Asia | 6.495243268 (5.127813515-8.359697877) |
| Oceania | 3.299971822 (2.217747187-5.866235273) |
| Australasia | 5.805910319 (5.207765695-6.448277889) |
| Central Asia | 6.814810274 (5.795822281-7.930922009) |
| High-income North America | 5.519439482 (5.150826205-5.741525631) |
| Western Europe | 5.326576393 (4.932300566-5.614404111) |
| High-income Asia Pacific | 12.6334374 (11.29380837-13.98455256) |
| Caribbean | 1.803171248 (1.56924967-2.06206047) |
| Southern Latin America | 1.764271587 (1.58886847-1.939599474) |
| Andean Latin America | 2.520114743 (1.94627967-3.159367051) |
| Tropical Latin America | 1.802184215 (1.685201706-1.896340795) |
| Central Latin America | 2.860623344 (2.591089395-3.152843749) |
| Central Sub-Saharan Africa | 5.090207052 (2.444983564-10.55668209) |
| North Africa and Middle East | 5.027182143 (4.285533351-5.817350396) |
| South Asia | 2.77401931 (2.498330553-3.060546423) |
| Latin America & Caribbean - WB | 2.230950193 (2.05996057-2.394806268) |
| Eastern Sub-Saharan Africa | 5.350588609 (4.125360025-6.988716237) |
| High SDI | 6.919252008 (6.385189258-7.312759155) |
| Low SDI | 5.239464337 (4.319982369-6.559449413) |
| Northern Africa | 8.408255686 (6.629766575-10.26435684) |
| High-middle SDI | 6.096897818 (5.228538665-7.174247304) |
| Middle SDI | 6.580064879 (5.720385415-7.791929322) |
| Low-middle SDI | 4.049216181 (3.683626436-4.475401506) |
| North America | 5.518608365 (5.15013339-5.74079455) |
| Africa | 7.184646327 (6.161739699-8.334829646) |
| Commonwealth Middle Income | 3.21394401 (2.921238057-3.522185576) |
| Southern Africa | 8.863712657 (7.068961461-11.19639891) |
| Southern Sub-Saharan Africa | 7.511641221 (6.446032141-8.732177827) |
| Western Sub-Saharan Africa | 8.74323112 (7.263401734-10.2134454) |
| Sub-Saharan Africa - WB | 6.820384443 (5.713471991-8.264241725) |
| East Asia & Pacific - WB | 9.553295257 (8.226646675-11.21448232) |
| Western Africa | 8.246015159 (6.874763755-9.728760088) |
| African Region | 6.5707061 (5.527935875-7.903929903) |
| Europe | 4.200037414 (3.929957957-4.404362325) |
| Eastern Mediterranean Region | 5.489338736 (4.732010791-6.273061831) |
| Region of the Americas | 3.761973908 (3.521424072-3.920085894) |
| Europe & Central Asia - WB | 4.212737754 (3.950122456-4.421169504) |
| America | 3.761973908 (3.521424072-3.920085894) |
| South Asia - WB | 2.773191896 (2.506524189-3.054951812) |
| Central Africa | 6.407067699 (4.509933645-9.465091132) |
| South-East Asia Region | 3.382316349 (2.98155447-3.88105875) |
| Middle East & North Africa - WB | 5.605174256 (4.665325839-6.523848665) |
| Commonwealth High Income | 5.878126974 (5.479081614-6.144276916) |
| Advanced Health System | 5.969103417 (5.513606533-6.27304233) |
| Limited Health System | 3.353976824 (3.007568196-3.72116381) |
| Commonwealth Low Income | 4.127215944 (3.11435333-5.455798101) |
| Western Pacific Region | 10.16410639 (8.682510622-11.96887711) |
| Eastern Africa | 4.028843563 (3.281736733-5.170507687) |
| Asia | 7.185529181 (6.333283285-8.278388927) |
| Minimal Health System | 10.72528772 (8.470716301-13.80557662) |
| Basic Health System | 7.310975388 (6.264977569-8.717709986) |
| European Region | 4.204950648 (3.940405025-4.411850926) |

Abbreviation: CI, confidence interval.

**Supplemental Table 3**

Global age-standardized DALY of liver cancer in 2021.

| Location | 2021 ASDR (1/100,000) R (95% CI) |
| --- | --- |
| Bangladesh | 60.77409007 (44.31779549-82.38243302) |
| Gabon | 276.14153 (168.9676122-426.7650162) |
| Palestine | 139.6995894 (110.5019323-175.0493186) |
| Kyrgyzstan | 90.20025495 (73.30676092-111.3022212) |
| North Macedonia | 194.1450017 (147.9830929-248.4034635) |
| Malaysia | 151.0706451 (119.2321936-188.158612) |
| Rwanda | 118.9926527 (80.41290568-170.4613218) |
| Zimbabwe | 405.8017149 (261.6997506-575.524483) |
| Oman | 91.30389255 (68.43277006-117.5398487) |
| Republic of Moldova | 81.48405906 (70.93659755-92.47969519) |
| Algeria | 46.94638225 (34.88532407-61.23801518) |
| Qatar | 317.7465057 (234.9424146-430.8001853) |
| India | 77.69872516 (68.93414473-87.82507953) |
| Burundi | 79.41370296 (50.21252107-131.3001737) |
| Somalia | 238.8090148 (122.4160528-433.4285264) |
| Benin | 358.5455171 (262.7524254-486.3161683) |
| Monaco | 181.4405371 (127.9340574-247.1961303) |
| Mauritania | 528.9775207 (278.290243-775.1991256) |
| Cambodia | 180.0845957 (97.59724549-319.7968913) |
| Azerbaijan | 215.9538982 (123.2922864-355.3846785) |
| Bahamas | 79.23283041 (63.52719724-99.39642687) |
| Estonia | 94.89188647 (79.13160389-113.5391523) |
| Fiji | 131.9180566 (90.04434137-182.6496954) |
| Greece | 102.3870454 (95.41465705-108.9799045) |
| Croatia | 68.39774537 (54.68151017-86.2279653) |
| Suriname | 70.6648205 (51.23888071-94.89611181) |
| Switzerland | 84.99017359 (76.25771799-94.58239146) |
| Mexico | 77.5153158 (68.13743164-87.15069576) |
| Belize | 61.29255486 (53.33796568-69.32468227) |
| Jordan | 36.49959975 (26.02945678-48.93447834) |
| Bolivia (Plurinational State of) | 86.14847597 (56.51257714-123.9004317) |
| Argentina | 29.19238611 (26.17189638-32.21037834) |
| Israel | 53.24437386 (47.63794497-59.13007185) |
| New Zealand | 101.7053918 (92.0220101-111.5042007) |
| Ireland | 71.470941 (62.22717117-79.73346698) |
| Kenya | 116.6058021 (89.88620472-156.5963801) |
| Panama | 77.41267978 (60.82364095-94.57949295) |
| Angola | 238.0666097 (61.7565964-599.3795215) |
| Sierra Leone | 267.252682 (183.676522-372.7397902) |
| Botswana | 164.1936732 (87.29699873-337.5010479) |
| Myanmar | 91.73231771 (47.73997833-178.6604198) |
| C么te d'Ivoire | 113.5042458 (69.38013211-169.8461229) |
| United Arab Emirates | 261.1152149 (193.7746413-346.5005594) |
| Puerto Rico | 74.73856188 (61.00714515-89.47776997) |
| Tajikistan | 115.678466 (73.34711789-171.9923767) |
| Venezuela (Bolivarian Republic of) | 66.47163966 (51.31827626-84.86140127) |
| American Samoa | 179.8524577 (138.9448567-230.727841) |
| Denmark | 62.36161273 (55.66527575-70.53289547) |
| Cuba | 38.04685798 (32.40754339-44.28974815) |
| Ukraine | 39.40885967 (28.25386181-51.26508256) |
| Haiti | 39.34770119 (21.46742663-73.83258066) |
| Armenia | 160.936638 (131.6959233-190.9394073) |
| Samoa | 130.4077284 (93.13674169-175.0739107) |
| Ghana | 222.0696583 (151.2531839-326.3117767) |
| Sri Lanka | 35.60638409 (22.94718401-50.59959115) |
| China | 239.9064603 (191.9778057-299.3726123) |
| Micronesia (Federated States of) | 147.5841001 (93.04888068-236.9491572) |
| Uzbekistan | 120.9533607 (87.85099232-158.6496416) |
| Guyana | 62.21451568 (46.43297095-80.06366683) |
| Poland | 44.24653829 (40.13320418-48.52533079) |
| San Marino | 53.2641288 (33.1712996-79.24746611) |
| Austria | 112.7908739 (100.6231218-125.1412237) |
| Greenland | 151.013718 (112.9728192-199.1201094) |
| Luxembourg | 89.37230628 (80.00559693-99.34813245) |
| Belarus | 74.43125635 (58.04570707-93.41867573) |
| Georgia | 109.1476474 (95.04429599-125.7095057) |
| Cook Islands | 287.5789815 (214.7429574-384.48157) |
| Peru | 59.1507877 (41.05015195-79.92451697) |
| Brazil | 47.83397773 (45.43595071-50.09742994) |
| Colombia | 51.10936911 (42.08010276-60.45614146) |
| Tonga | 596.987822 (413.3030531-832.7044691) |
| Uruguay | 59.32877189 (50.94154326-67.66702207) |
| Australia | 125.9011773 (112.9566623-140.1185331) |
| Netherlands | 58.79457921 (53.75402108-64.34135657) |
| Guinea | 569.8733761 (369.5433097-844.2151188) |
| Cyprus | 68.60062198 (50.99988883-92.38590227) |
| Thailand | 294.6620274 (218.2686292-402.9631971) |
| Mauritius | 18.52541587 (16.95909137-19.72661399) |
| Canada | 103.9328508 (93.5646344-115.2016854) |
| Dominican Republic | 51.44632831 (37.38328827-68.54168568) |
| Brunei Darussalam | 243.0389483 (189.3787756-313.4407007) |
| Democratic Republic of the Congo | 99.96112322 (49.80761703-211.4295954) |
| Costa Rica | 136.464551 (117.8146274-157.220812) |
| Kiribati | 178.3758513 (126.9294183-251.9207437) |
| Libya | 200.8388101 (144.2513662-274.4587108) |
| Slovakia | 94.57313088 (67.78788833-130.9044737) |
| Latvia | 82.71629696 (68.56915143-98.18276045) |
| Serbia | 123.6035325 (91.21788759-162.5217742) |
| Tokelau | 136.0231702 (86.53270175-210.1756265) |
| Seychelles | 111.556826 (89.4459541-139.2755197) |
| Iceland | 90.19380398 (78.99035637-102.02848) |
| Trinidad and Tobago | 56.83964678 (43.16134151-72.36345592) |
| Taiwan, China | 254.9262394 (228.367104-285.9808933) |
| South Africa | 183.1648848 (157.8750921-213.2523813) |
| Viet Nam | 346.8410068 (246.6638686-499.2643961) |
| Turkmenistan | 151.3696176 (113.7729249-203.3222224) |
| Bosnia and Herzegovina | 143.0287332 (106.1452898-178.0933269) |
| Liberia | 492.3254299 (321.3573336-699.9249818) |
| Republic of Korea | 354.5667156 (291.3399569-435.3092645) |
| United States Virgin Islands | 65.28077362 (43.98001482-95.2018662) |
| Bahrain | 109.6413897 (83.70591585-142.798364) |
| France | 137.8872438 (120.4111635-155.9550714) |
| Guatemala | 131.8947751 (113.2631523-152.3348303) |
| Nepal | 83.96089071 (53.36147801-120.7382996) |
| Vanuatu | 120.2369221 (77.87244964-192.3782088) |
| South Sudan | 187.75564 (120.9777939-273.5670949) |
| Chad | 381.1477723 (231.1735664-637.9176267) |
| Spain | 123.7217754 (109.3646037-138.6095738) |
| Belgium | 90.51705063 (81.64241408-100.5256985) |
| Northern Mariana Islands | 174.9900595 (143.5946791-213.2473005) |
| Malta | 64.161281 (54.75741589-74.99878973) |
| Nicaragua | 77.14284084 (58.05408522-102.1114135) |
| Iran (Islamic Republic of) | 68.49629891 (60.59790205-76.996135) |
| Solomon Islands | 140.878645 (92.6069634-227.7070346) |
| United Republic of Tanzania | 131.6705525 (92.86641492-190.3210422) |
| Comoros | 130.2437286 (87.64360343-190.3142235) |
| Saudi Arabia | 145.9715118 (107.4797848-193.6156876) |
| Saint Lucia | 41.22659356 (33.19656203-49.84815084) |
| Mali | 795.8078783 (584.8081641-1063.477311) |
| United States of America | 106.2407322 (101.1222674-109.9996758) |
| Gambia | 977.0174414 (625.5506855-1453.285663) |
| Bhutan | 107.8475358 (69.09904732-153.1501144) |
| Lesotho | 481.7962705 (203.2975249-1072.224795) |
| Bermuda | 35.5322531 (29.867011-43.14304395) |
| Zambia | 134.3899544 (60.08966375-309.6168013) |
| Burkina Faso | 512.9880326 (255.4298516-887.150167) |
| Tunisia | 49.82164557 (33.47628777-69.89994826) |
| Norway | 82.31936423 (76.92326476-87.26640056) |
| Sao Tome and Principe | 65.25103615 (39.64854422-111.2352537) |
| Eritrea | 107.4976814 (70.73459606-176.7318096) |
| Cabo Verde | 336.3303445 (227.6824444-450.6716086) |
| Nauru | 180.4340016 (112.8219184-272.5518648) |
| Niger | 254.1831147 (166.7860975-410.8227437) |
| Bulgaria | 91.47404895 (68.35535017-122.2907271) |
| Sudan | 94.38589384 (62.59975986-135.8585969) |
| Iraq | 119.3254538 (84.86391344-157.5577852) |
| Barbados | 59.43437126 (45.98479626-75.52205045) |
| Czechia | 59.28054607 (47.19968539-72.01577091) |
| Kuwait | 25.57504387 (20.47436581-31.11346953) |
| Ethiopia | 69.92987066 (47.58677166-111.4312911) |
| United Kingdom | 121.8715936 (116.3878563-126.0056728) |
| Indonesia | 108.265132 (63.72143342-170.0378462) |
| Togo | 220.7982558 (146.4052089-342.398368) |
| Saint Kitts and Nevis | 82.86447132 (67.73565861-96.91609543) |
| Central African Republic | 212.2915273 (96.41110832-421.1432976) |
| Madagascar | 78.52434287 (52.135136-120.2423971) |
| Grenada | 94.11848065 (80.41024369-108.0965105) |
| Turkiye | 69.64166948 (54.45914929-88.93602606) |
| Romania | 77.38135355 (64.43069458-93.32644889) |
| Philippines | 173.6022762 (141.8280622-210.2255622) |
| Yemen | 58.3630822 (34.02616433-102.0213599) |
| Democratic People's Republic of Korea | 211.1282138 (134.3628266-301.8961822) |
| Timor-Leste | 77.94975743 (48.94505612-127.2454901) |
| Slovenia | 103.5331786 (85.49376101-126.6615597) |
| Japan | 145.7437487 (133.925062-153.1291309) |
| Finland | 90.13062189 (80.78095058-99.82350601) |
| Portugal | 119.3529128 (107.3178762-133.7641106) |
| El Salvador | 42.7306202 (33.45898482-53.71632554) |
| Singapore | 126.5236142 (113.9718615-138.952481) |
| Germany | 95.30940304 (87.36298743-104.1101158) |
| Honduras | 91.56334204 (67.73221749-126.7093502) |
| Sweden | 65.8011877 (57.00710491-76.08166806) |
| Lao People's Democratic Republic | 189.3101944 (127.6833705-263.3478666) |
| Kazakhstan | 126.6308109 (105.0588976-147.9836454) |
| Marshall Islands | 111.721726 (66.16187308-192.5860907) |
| Lithuania | 88.57217499 (75.3275836-103.5853727) |
| Maldives | 104.3966265 (75.23962729-137.4554838) |
| Jamaica | 40.57713493 (30.53785792-52.00797649) |
| Papua New Guinea | 71.77672694 (34.09264453-176.8801677) |
| Mongolia | 1993.245112 (1531.377315-2547.245191) |
| Italy | 97.71271168 (90.54452595-104.1401198) |
| Saint Vincent and the Grenadines | 72.26314463 (62.74866819-82.77778641) |
| Russian Federation | 70.19554811 (64.5450364-76.09163373) |
| Andorra | 232.4847573 (158.2949579-323.8409604) |
| Chile | 73.29744057 (65.60640048-81.10870046) |
| Paraguay | 80.59430359 (56.67646694-107.6984789) |
| Ecuador | 67.75528743 (53.04972456-86.04756315) |
| Uganda | 206.6421369 (148.6298746-284.5472623) |
| Namibia | 89.22386812 (64.56100348-122.7661819) |
| Eswatini | 597.2657083 (232.1811914-1269.656783) |
| Syrian Arab Republic | 127.4299135 (90.64886576-170.7585442) |
| Guam | 167.8888094 (140.0123056-195.3847095) |
| Pakistan | 110.3013897 (82.74397833-142.5760304) |
| Cameroon | 346.7024661 (195.3776356-576.8376697) |
| Afghanistan | 181.1893375 (129.050478-250.0374725) |
| Antigua and Barbuda | 64.162267 (59.7874964-69.04154162) |
| Congo | 204.8203728 (103.4925485-390.4023713) |
| Equatorial Guinea | 154.0068919 (92.76637067-245.2399267) |
| Albania | 191.346059 (128.1944586-283.1417238) |
| Egypt | 476.5769208 (368.5978471-598.290036) |
| Guinea-Bissau | 553.7424467 (372.8403856-757.3389512) |
| Dominica | 86.26201856 (65.31469425-113.5248706) |
| Lebanon | 58.08568743 (44.42416595-73.91790359) |
| Hungary | 43.71645375 (34.83064826-53.89346457) |
| Morocco | 14.28958232 (10.11190083-18.4612887) |
| Montenegro | 154.8023418 (112.6828711-208.4843347) |
| Nigeria | 124.3442722 (85.7122722-177.1795844) |
| Senegal | 256.3215335 (182.796504-349.5604288) |
| Tuvalu | 135.343783 (91.76521468-195.4193542) |
| Djibouti | 123.2290211 (80.34738492-187.7457977) |
| Malawi | 145.4917963 (106.5319843-197.0370952) |
| Mozambique | 592.2429263 (324.3897054-1119.78852) |
| Niue | 139.9679824 (93.3054107-211.4408321) |
| Palau | 229.1548827 (164.6824045-311.5950185) |
| South Asia | 79.6907074 (71.78139034-88.0310274) |
| Central Europe | 73.69665681 (65.97284093-82.12675141) |
| Central Asia | 188.7727065 (160.0232902-221.6457386) |
| Eastern Europe | 64.40365998 (59.67355811-69.67472198) |
| Low SDI | 151.065682 (122.3814229-192.7778512) |
| Eastern Sub-Saharan Africa | 150.4170079 (113.9617926-198.6240404) |
| High-income North America | 106.0361475 (100.5914513-109.9514988) |
| Central Latin America | 73.56926586 (66.47251667-81.22013984) |
| Australasia | 121.9936717 (109.818048-135.4587843) |
| Tropical Latin America | 48.61544194 (46.02540833-50.99580305) |
| Southern Latin America | 44.04878362 (39.67804863-48.34099615) |
| High-income Asia Pacific | 205.8485454 (185.2515885-229.3065391) |
| Andean Latin America | 65.791162 (50.5564346-82.91578266) |
| Latin America & Caribbean - WB | 58.51072795 (54.16083163-62.9484863) |
| Europe & Central Asia - WB | 93.08685701 (87.95611198-97.60361631) |
| Sub-Saharan Africa - WB | 193.7984212 (159.4378783-240.0514701) |
| Southern Sub-Saharan Africa | 218.1948803 (185.22997-257.1864662) |
| Low-middle SDI | 116.3749713 (104.9089509-128.9685694) |
| African Region | 187.201726 (154.5115454-230.7225919) |
| Southern Africa | 250.5578925 (194.542926-322.9581285) |
| Northern Africa | 228.6082383 (178.4126156-280.7094976) |
| Commonwealth Middle Income | 92.58351509 (83.83336858-102.0596297) |
| High-middle SDI | 152.6041409 (129.4886197-181.8067697) |
| Western Africa | 235.3002452 (192.8862149-281.0100436) |
| East Asia & Pacific - WB | 226.3624828 (193.1710526-271.4837358) |
| Asia | 171.4663844 (150.0513432-198.8573687) |
| High SDI | 127.9176349 (120.5725145-134.4056199) |
| Western Europe | 106.0745608 (99.82499272-111.4183203) |
| Western Pacific Region | 239.2239361 (201.4738022-285.9006615) |
| Oceania | 96.36736419 (63.68862792-175.1174296) |
| North Africa and Middle East | 134.7875099 (114.0801901-156.584802) |
| Western Sub-Saharan Africa | 249.9253401 (204.2169257-298.725049) |
| Central Sub-Saharan Africa | 143.530179 (69.1425337-297.6104443) |
| Southeast Asia | 179.6697621 (141.2177013-234.2645895) |
| Middle East & North Africa - WB | 149.2780037 (123.2310608-175.4682445) |
| Caribbean | 48.3789962 (41.47056354-55.87919774) |
| South Asia - WB | 79.78762491 (71.9773891-88.15674207) |
| Commonwealth High Income | 116.2106519 (110.0747262-121.4090749) |
| Global | 149.2867379 (135.2351823-167.4849248) |
| Commonwealth Low Income | 119.5792391 (88.94504833-162.3357258) |
| Region of the Americas | 80.38494434 (76.09336973-83.90749023) |
| North America | 106.0163211 (100.5732463-109.9303085) |
| Eastern Africa | 115.0590287 (92.35113879-150.4613701) |
| South-East Asia Region | 94.45271496 (83.30199885-108.7142758) |
| Middle SDI | 169.7104805 (147.4078391-200.8529459) |
| Europe | 91.43261997 (86.47933577-95.78964074) |
| Africa | 202.1752642 (170.5313802-238.2619841) |
| European Region | 92.96231313 (87.8832979-97.48524536) |
| East Asia | 239.8063469 (193.3388151-296.8288246) |
| America | 80.38494434 (76.09336973-83.90749023) |
| Eastern Mediterranean Region | 150.2298971 (128.877846-173.1427774) |
| Advanced Health System | 114.9114706 (108.5870567-120.251809) |
| Basic Health System | 187.632756 (160.4256263-224.6770396) |
| Central Africa | 182.0738056 (126.3232945-274.5111195) |
| Limited Health System | 96.87214368 (86.23872813-109.1206665) |
| Minimal Health System | 302.4917759 (236.2071795-395.5000713) |

Abbreviation: CI, confidence interval.
